# Keeping *Candida* commensal: how lactobacilli antagonize pathogenicity of *Candida albicans* in an *in vitro* gut model

**DOI:** 10.1242/dmm.039719

**Published:** 2019-09-01

**Authors:** Katja Graf, Antonia Last, Rena Gratz, Stefanie Allert, Susanne Linde, Martin Westermann, Marko Gröger, Alexander S. Mosig, Mark S. Gresnigt, Bernhard Hube

**Affiliations:** 1 Department of Microbial Pathogenicity Mechanisms, Leibniz Institute for Natural Product Research and Infection Biology–Hans-Knoell-Institute, Beutenbergstraße 11A, 07745 Jena, Germany; 2 Center for Electron Microscopy Jena University Hospital, Ziegelmühlenweg 1, 07743 Jena, Germany; 3 Center for Sepsis Control and Care (CSCC), University Hospital Jena, Am Klinikum 1, 07747 Jena, Germany; 4 Institute of Biochemistry II, Jena University Hospital, Am Klinikum 1, 07747 Jena, Germany; 5 Friedrich Schiller University, Fürstengraben 1, 07743 Jena, Germany

**Keywords:** *Candida albicans*, Microbiota, Commensalism, Lactobacilli, Antagonism, *In vitro* model

## Abstract

The intestine is the primary reservoir of *Candida albicans* that can cause systemic infections in immunocompromised patients. In this reservoir, the fungus exists as a harmless commensal. However, antibiotic treatment can disturb the bacterial microbiota, facilitating fungal overgrowth and favoring pathogenicity. The current *in vitro* gut models that are used to study the pathogenesis of *C. albicans* investigate the state in which *C. albicans* behaves as a pathogen rather than as a commensal. We present a novel *in vitro* gut model in which the fungal pathogenicity is reduced to a minimum by increasing the biological complexity. In this model, enterocytes represent the epithelial barrier and goblet cells limit *C. albicans* adhesion and invasion. Significant protection against *C. albicans-*induced necrotic damage was achieved by the introduction of a microbiota of antagonistic lactobacilli. We demonstrated a time-, dose- and species-dependent protective effect against *C. albicans*-induced cytotoxicity. This required bacterial growth, which relied on the presence of host cells, but was not dependent on the competition for adhesion sites. *Lactobacillus rhamnosus* reduced hyphal elongation, a key virulence attribute. Furthermore, bacterial-driven shedding of hyphae from the epithelial surface, associated with apoptotic epithelial cells, was identified as a main and novel mechanism of damage protection. However, host cell apoptosis was not the driving mechanism behind shedding. Collectively, we established an *in vitro* gut model that can be used to experimentally dissect commensal-like interactions of *C. albicans* with a bacterial microbiota and the host epithelial barrier. We also discovered fungal shedding as a novel mechanism by which bacteria contribute to the protection of epithelial surfaces.

This article has an associated First Person interview with the joint first authors of the paper.

## Abstract (precis)

**Editor's choice:** Using antagonistic lactobacilli, *C. albicans* pathogenicity was reduced to a minimum in an *in vitro* gut model consisting of enterocytes and goblet cells.

## INTRODUCTION

The gut epithelium is a barrier between the sterile host environment and gut microbiota. Intestinal epithelial cells (IECs) represent the first line of defense against microbial invasion by being a passive physical barrier that prevents translocation (Peterson and Artis, 2014). In addition, goblet cells within the intestinal epithelium produce a protective mucus layer (Maynard et al., 2012; Yan et al., 2013). This layer serves as an anchor for the attachment of microbes and represents a nutrient source for mutualistic bacteria living within the gut (Cockburn and Koropatkin, 2016) that produce metabolites, nourishing IECs (Maynard et al., 2012). The gut microbiota is crucial for the development and maintenance of mucosal host defense by improving the physical barrier, competition for nutrients and adhesion sites with potential pathogens, and by developing the immune system (Sekirov et al., 2010; Bischoff et al., 2014). However, when the balance of the microbiota is impaired, opportunistic pathogens may outgrow the beneficial microbiota. The host's immune system and a functional intestinal barrier are generally sufficient to prevent infection. However, cytostatic therapy for the treatment of cancer targets fast-dividing cells. As a result, not only malignant cells are targeted, but also cells of the immune system and intestinal epithelial lining. In this immunocompromised state, patients are predisposed to develop opportunistic infections with otherwise harmless commensals of the microbiota. For example, the yeast *Candida albicans* exists as a commensal in the gastrointestinal tract of approximately 50% of the western population (Bougnoux et al., 2006), but can cause severe systemic infections under certain predisposing factors. Use of broad-spectrum antibiotics and a compromised immune status are such factors that can lead to *C. albicans* overgrowth and a switch from commensalism to pathogenicity (Bassetti et al., 2011; Mason et al., 2012), potentially resulting in translocation through the intestinal barrier and disseminated infections (Koh et al., 2008). Indeed, the main reservoir of *C. albicans* that causes systemic candidiasis is the gut (Gouba and Drancourt, 2015; Miranda et al., 2009; Nucci and Anaissie, 2001).

The association between candidiasis and the use of broad-spectrum antibiotics is believed to relate to the eradication of bacteria that antagonize *C. albicans* pathogenicity. Although numerous bacterial species interact with *C. albicans* (Bamford et al., 2009; Cruz et al., 2013; Fan et al., 2015; Förster et al., 2016), *Lactobacillus* species are the most widely known for their antagonistic potential. Most studies that aimed at investigating the mechanisms by which lactobacilli can counteract *C. albicans* were performed in host-free environments (Köhler et al., 2012; Strus et al., 2005) or on human (vaginal, oral or cervical) epithelial cells (do Carmo et al., 2016; Donnarumma et al., 2014; Mailänder-Sánchez et al., 2017; Rizzo et al., 2013). Lactobacilli have been shown to counteract *C. albicans* through inhibition of fungal growth (Coman et al., 2015; de Barros et al., 2018; Hasslöf et al., 2010; Köhler et al., 2012; Ribeiro et al., 2017; Strus et al., 2005), inhibition of hyphal morphogenesis (Allonsius et al., 2019), prevention of adhesion (Donnarumma et al., 2014; Mailänder-Sánchez et al., 2017; Rizzo et al., 2013), competition for nutrients (Mailänder-Sánchez et al., 2017) or by influencing immune responses (Marranzino et al., 2012; Plantinga et al., 2012; Rizzo et al., 2013). Among other lactobacilli, *L. rhamnosus* can reduce *C. albicans* hyphal induction and biofilm formation via cell-cell interactions and the secretion of exometabolites (James et al., 2016; Matsubara et al., 2016a). Exopolysaccharides of *L. rhamnosus* GG interfere with hyphal formation and adhesion to vaginal and bronchial epithelial cells (Allonsius et al., 2017). *L. rhamnosus* GG also protects oral epithelial cells against *C. albicans-*induced damage by competing for adhesion sites as well as depleting nutrients (Mailänder-Sánchez et al., 2017)*.* Furthermore, lactobacilli can produce compounds such as hydrogen peroxide, lactic acid, bacteriocins and biosurfactants, which inhibit the growth of potential pathogens (reviewed by Förster et al., 2016 and Matsubara et al., 2016b). The importance of the microbiota in preventing damage to epithelial cells by *C. albicans* is demonstrated by the fact that any epithelial cell layer exposed to *C. albicans in vitro* is rapidly and efficiently invaded and damaged via necrotic cell death and unable to prevent translocation in the absence of a microbiota (Allert et al., 2018).

Here, we studied whether we could achieve a ‘commensal’ model in which the gut epithelial barrier is subjected to minimal *C. albicans-*induced damage and translocation by increasing biological complexity. Using a model consisting of intestinal epithelial cells, mucus-producing goblet cells and lactobacilli, we investigated how lactobacilli antagonize *C. albicans*-induced necrotic cytotoxicity.

## RESULTS

### *C. albicans*-induced epithelial damage is reduced by colonization with *Lactobacillus* species

**Fig. 1.**
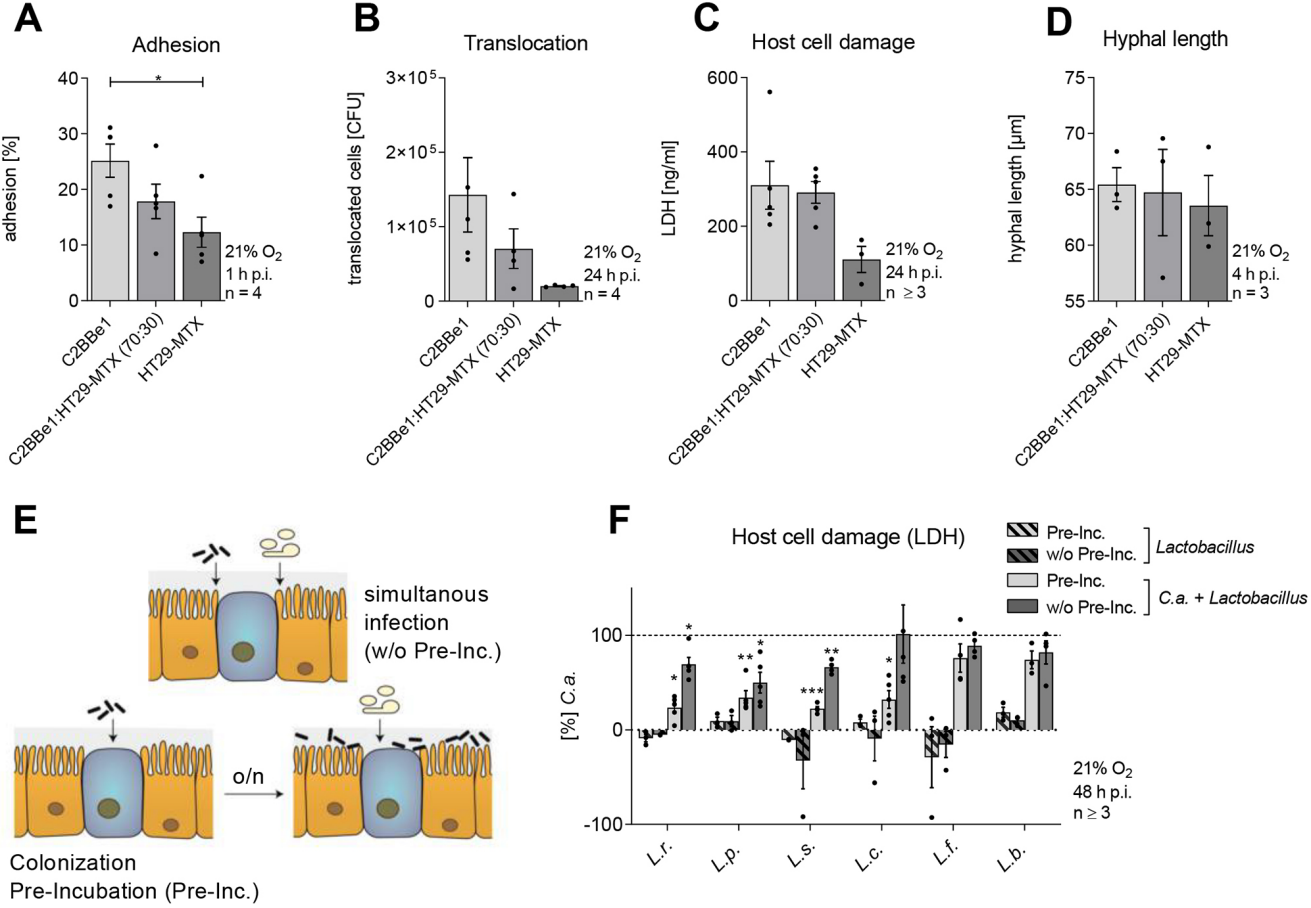
**Influence of combined host cell types on *C. albicans* adhesion, translocation and cytotoxicity and the protective effect of *Lactobacillus* species.** (A-D) Percentage of *C. albicans* adhered to IECs at 1 h post-infection (A), translocation of *C. albicans* across IECs at 24 h post-infection (B), LDH release of IECs at 24 h post-infection (C) or *C. albicans* hyphal length at 4 h post-infection (D) on C2BBe1 (enterocyte) and HT29-MTX (mucus-secreting goblet cell) monocultures or in co-culture (C2BBe1:HT29-MTX). (E) Schematic of the two different infection regimens. IECs C2BBe1 (orange) and HT29-MTX (blue) were infected with *C. albicans* (white) and lactobacilli (black) simultaneously (w/o Pre-Inc.) or IECs were colonized (Pre-Inc.) with lactobacilli 18 h (o/n) before *C. albicans* infection. (F) LDH release of IECs colonized or not with *L. paracasei* (*L.p*.), *L. rhamnosus* (*L.r*.), *L. salivarius* (*L.s*.), *L. fermentum* (*L.f*.) or *L. brevis* (*L.b*.) (MOI 50) 48 h post *C. albicans* (*C.a.*) infection (MOI 1). Results were normalized to *C. albicans* single infection. Data are mean±s.e.m. *P<0.05, **P<0.01, ***P<0.005 (*t*-test).

Our study aimed to establish an *in vitro* model, which mimics the commensal phase of *C. albicans* in the gut, in order to dissect commensal-like scenarios. First, we reduced *C. albicans-*induced damage to a minimum by manipulating the composition of the epithelial barrier. As mucus can dampen virulence attributes of *C. albicans* (Kavanaugh et al., 2014), the mucus-producing goblet cell line HT29-MTX, which has been extensively validated for compatibility and functional properties with C2BBe1 enterocytes (Ferraretto et al., 2018), was introduced in an existing model for *Candida*-gut translocation (Allert et al., 2018). In comparison to C2BBe1 enterocytes alone, the presence of HT29-MTX cells, at a ratio of 70:30 respectively, reduced adhesion and translocation of *C. albicans* by approximately 30% (Fig. 1A,B). However, the potential for *C. albicans* necrotic cell damage, as determined by the concentration of epithelial cytosolic lactate dehydrogenase (LDH) in the culture supernatants (Chan et al., 2013), as well as *C. albicans*’ hyphal length, remained comparable to the C2BBe1 monoculture model (Fig. 1C,D).

To introduce another level of complexity, an artificial ‘microbiota’ was included. Various *Lactobacillus* species with known *Candida-*antagonizing potential were investigated for their impact on *C. albicans* pathogenicity. The gut model was either colonized (pre-incubated; Pre-Inc.) with lactobacilli overnight followed by fungal infection or simultaneously colonized during *C. albicans* infection (w/o Pre-Inc.) (Fig. 1E). Colonization with *Lactobacillus* species alone did not induce necrotic epithelial cell damage (Fig. 1F). However, colonization with *L. paracasei*, *L. rhamnosus*, *L. salivarius* or *L. casei* before *C. albicans* infection reduced *C. albicans*-induced necrotic cytotoxicity (Fig. 1F). Colonization with *L. fermentum* and *L. brevis* or simultaneous colonization of lactobacilli with *C. albicans* infection did not influence *Candida*-induced damage. As lactobacilli were successful in protecting the epithelial barrier, we systematically investigated the factors that might contribute to this effect.

### *Lactobacillus*-mediated damage protection is dose-, time- and species dependent

**Fig. 2.**
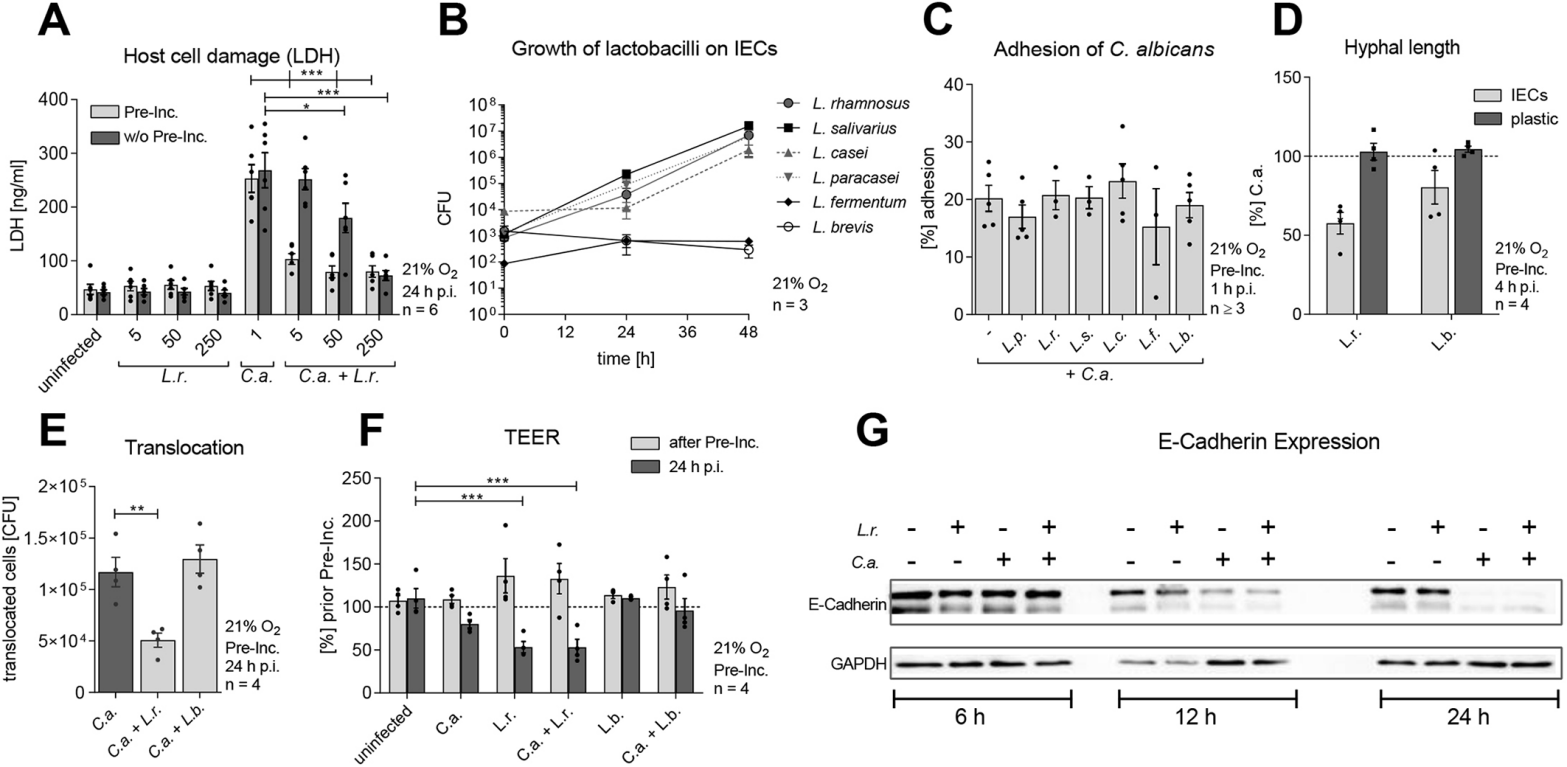
**Growth of lactobacilli on IECs and their influence towards *C. albicans* cytotoxicity, adhesion, hyphal length and translocation.** (A) LDH release of IECs colonized (Pre-Inc.) or simultaneously colonized (w/o Pre-Inc.) with *L. rhamnosus* (*L.r*.) at different MOI (5, 50 or 250) and infected or not with *C. albicans* (*C.a.*) (MOI 1) and measured at 24 h post-infection. (B) Growth of *L. paracasei* (*L.p*.), *L. rhamnosus*, *L. salivarius* (*L.s*.), *L. fermentum* (*L.f*.), and *L. brevis* (*L.b*.) on IECs. (C) Percentage of *C. albicans* adhered to IECs colonized with different *Lactobacillus* species (MOI 50) at 1 h post-infection. (D) *C. albicans* hyphal induction on IECs or on plastic colonized with *L. rhamnosus* or *L. brevis* (MOI 50) at 4 h post-infection. Results were normalized to *C. albicans* single infection. (E) Translocation of *C. albicans* (MOI 1) across IECs colonized with *L. rhamnosus* or *L. brevis* (MOI 50) at 24 h post-infection. (F) Assessment of epithelial barrier integrity measured as the loss of transepithelial electrical resistance (TEER) in response to *L. rhamnosus* or *L. brevis* (MOI 50) colonization and *C. albicans* infection in the presence or absence of *Lactobacillus* colonization at 24 h post-infection. Data are TEER loss in percentage of uninfected host cells (before pre-incubation). (G) E-Cadherin protein expression analyzed by western blot compared to GAPDH in IECs that were left uninfected or colonized with *L. rhamnosus* (MOI 50) and infected with *C. albicans* for 6, 12 and 24 h. Data are mean±s.e.m. *P<0.05, **P<0.01, ***P<0.005 (*t*-test).

When colonized during *C. albicans* infection, *L. rhamnosus* dose-dependently reduced damage (Fig. 2A). A 250-fold excess of bacteria to *Candida* cells decreased LDH release almost to the degree of uninfected epithelial cells, yet colonization of IECs before *C. albicans* infection required significantly lower numbers of bacteria (5- to 50-fold excess of bacterial cells) to achieve damage protection. This may be because of the capacity of *L. rhamnosus* to multiply during colonization (Fig. 2B), meaning that a 50-fold excess of bacterial cells is sufficient to reduce *C. albicans*-induced damage when time for replication is given and the bacterium is able to grow on host cells. Conversely, the inability of *L. fermentum* and *L. brevis* to protect against *Candida*-induced damage (Fig. 1F) correlated with their inability to replicate in the model (Fig. 2B). Therefore, *Lactobacillus*-mediated damage protection relies on the ability of the bacteria to proliferate and an interaction of a high quantity of bacteria with *C. albicans*. In subsequent experiments, colonization of 50 bacterial cells per yeast cell was used to study the protective effects mediated by lactobacilli.

### Lactobacilli do not interfere with fungal adhesion, but suppress filamentation and translocation

Prerequisite processes for *C. albicans* pathogenicity are adhesion to host cells and a subsequent morphological transition from yeast to hyphae, enabling the fungus to invade host cells, cause damage and translocate across barriers (Allert et al., 2018; Dalle et al., 2010). Therefore, the impairment of *C. albicans* adhesion and/or filamentation could be a potential mechanism for damage protection by lactobacilli (Allonsius et al., 2017; Coman et al., 2015; Donnarumma et al., 2014; James et al., 2016; Kang et al., 2018; Niu et al., 2017; Parolin et al., 2015; Santos et al., 2016; Tan et al., 2017; Wang et al., 2017). Although colonization of the model by *Lactobacillus* species did not interfere with *C. albicans*’ adhesion to IECs and hyphal formation per se (Fig. 2C), *L. rhamnosus* significantly reduced hyphal length (42% reduction in length) (Fig. 2D). *L. brevis*, as a less-protective *Lactobacillus* species, did not reduce hyphal length. Interestingly, lactobacilli only grew in the host cell culture medium when host cells were present. Therefore, they did not reach the necessary number of bacterial cells to gain influence on fungal filamentation in the absence of host cells. As a result, *Lactobacillus-*mediated protection relied on host cells to sustain bacterial growth. The reduced *C. albicans* filamentation in the presence of *L. rhamnosus* was accompanied by reduced translocation (Fig. 2E)*.* Consequently, *L. brevis*, which did not reduce filamentation, also failed to reduce *C. albicans* translocation.

Despite reduced hyphal length and translocation, a loss of epithelial barrier integrity [quantified via measurement of transepithelial electrical resistance (TEER)] was observed in both scenarios: *C. albicans* single infection and when the model was colonized with *L. rhamnosus* (Fig. 2F). Even *L. rhamnosus* alone decreased epithelial integrity after 24 h of colonization*. C. albicans-*induced loss of epithelial integrity is accompanied by decreasing epithelial E-cadherin (CDH1) levels induced by increasing levels of µ-calpain (CAPN1), a proteolytic enzyme that targets E-cadherin (Rouabhia et al., 2012; Xu et al., 2016). E-cadherin degradation was observed when IECs were infected with *C. albicans* for at least 12 h (Fig. 2G). Although *L. rhamnosus* reduced fungal translocation, E-cadherin breakdown still occurred. Nevertheless, stable E-cadherin levels during *L. rhamnosus* colonization indicate that the bacteria do not affect the epithelial E-cadherin level.

### Damage protection by lactobacilli is associated with reduced IEC damage and stress response

For oral, vaginal and intestinal epithelial cells, *Candida*-induced damage correlates with the induction of mitogen-activated protein kinase (MAPK) and NF-κB responses (Böhringer et al., 2016; Moyes et al., 2011, 2010). Therefore, key mediators of this ‘danger’ response were assessed for their activation during the *Lactobacillus*-*Candida* interaction on IECs.

**Fig. 3.**
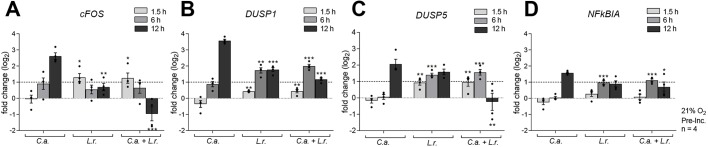
**Lactobacilli suppress the expression of damage- and stress-related host factors by *C. albicans*.** (A-D) The relative mRNA expression of *cFOS* (A), *DUSP1* (B), *DUSP5* (C) and *NFKBIA* (D) in IECs either left untreated or colonized with *L. rhamnosus* (*L.r.*) (MOI 50) and subsequently infected or not with *C. albicans* (*C.a.*) (MOI 1) for 1.5, 6 or 12 h. Expression levels were normalized to the reference genes *ACT1* and *GAPDH*. Data are mean±s.e.m. *P<0.05, **P<0.01, ***P<0.005 (one-way ANOVA).

The *cFOS* gene, which encodes a subunit of the heterodimeric transcription factor AP-1, was highly expressed in IECs 12 h after *Candida* infection (Fig. 3A). The dual-specificity phosphatases are primary targets of AP-1 (Patterson et al., 2009) and inactivate stress-related MAPKs such as p38, ERK and JNK. This provides a negative feedback on MAPK activation. The *DUSP1* and *DUSP5* genes, encoding two of these phosphatases, were upregulated by *C. albicans* infection (Fig. 3B,C). Activation of the NF-κB signaling pathway induces expression of several NF-κB transcriptional target genes, including NFKB inhibitor alpha (*NFKBIA*), which functions as a negative feedback regulator of NF-κB activation (Jacobs and Harrison, 1998). *NFKBIA* gene expression was observed 12 h after *Candida* infection (Fig. 3D). *L. rhamnosus* colonization alone or combined with *C. albicans* infection resulted in a marginal induction of *cFOS*, *DUSP1*, *DUSP5* and *NFKBIA* expression at early time points, but *L. rhamnosus* colonization before infection downregulated these genes at later stages of infection. These data indicate that *L. rhamnosus*, on a molecular level, counteracts the damage response to *C. albicans*.

### *Lactobacillus*-mediated damage protection is independent of oxygen levels

**Fig. 4.**
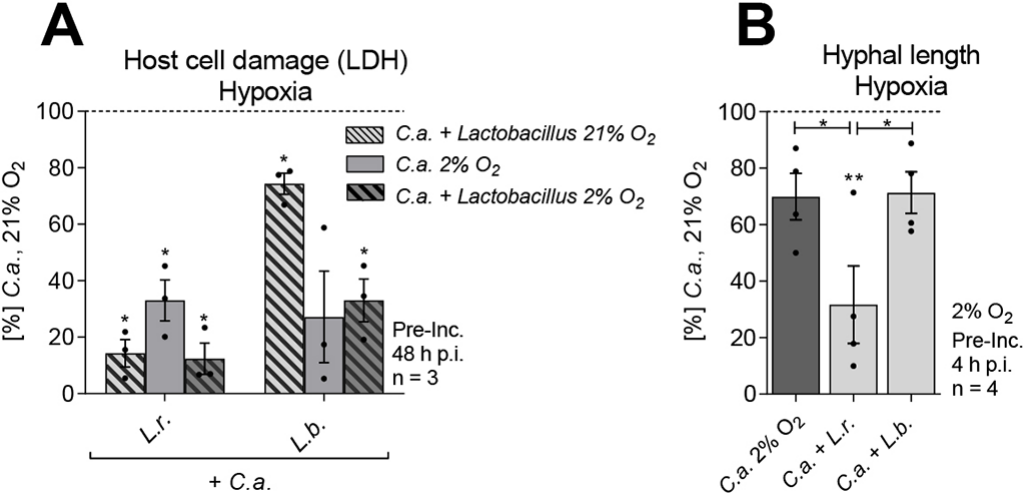
**The protective effect of lactobacilli under hypoxia.** (A) LDH release of IECs colonized with *L. rhamnosus* (*L.r.*) and *L. brevis* (*L.b.*) (MOI 50) and infected or not with *C. albicans* (*C.a.*) (MOI 1) at 48 h post-infection at an oxygen level of 21% or 2%. (B) Hyphal length of *C. albicans* on IECs colonized with *L. rhamnosus* or *L. brevis* (MOI 50) at 4 h post-infection. Results were normalized to *C. albicans* single infection at 21% O_2_. Data are mean±s.e.m. *P<0.05, **P<0.01 compared with *C. albicans* single infection at 21% oxygen (*t*-test).

As the gastrointestinal tract has distinct hypoxic niches, with low oxygen tension in the intestinal lumen and higher oxygen tension towards the crypts (Zheng et al., 2015), hypoxic conditions were applied to further work towards a model in which *C. albicans* exhibits a commensal-like co-existence. As lactobacilli preferably grow under anaerobic conditions, we speculated that hypoxia might augment *Lactobacillus*-mediated damage protection. A minimal oxygen level of 2% was selected, as lower levels reduced IEC viability. Compared to 21%, 2% oxygen diminished *C. albicans*-induced cytotoxicity by more than 50% (Fig. 4A). This correlated with reduced hyphal formation during hypoxia (Fig. 4B), which was further reduced when the model was colonized with *L. rhamnosus*. Protection against *C. albicans-*induced cytotoxicity was more pronounced in the presence of protective *Lactobacillus* species; however, not significantly different from damage protection at 21% oxygen. To further investigate the mechanism associated with *Lactobacillus*-mediated damage protection, experiments were continued at 21% oxygen.

### *Lactobacillus*-mediated damage protection is contact-dependent

**Fig. 5.**
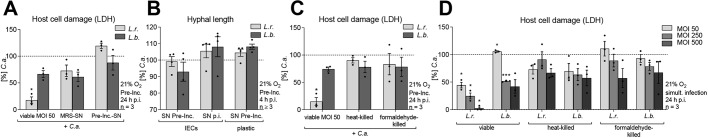
**Damage protection requires the presence of viable lactobacilli.** (A) LDH release at 24 h post-infection of IECs either colonized with viable *L. rhamnosus* (*L.r*.) or *L. brevis* (*L.b.*) (MOI 50), co-incubated with MRS supernatant (MRS-SN) or colonized (Pre-Inc. SN) (see Materials and Methods) and infected with *C. albicans* (*C.a.*) (MOI 1). The results were normalized to *C. albicans* single infection. (B) *C. albicans* hyphal length after 4 h of infection on IECs or on plastic co-incubated with Pre-Inc. supernatant (SN Pre-Inc.) or post-infection supernatant (SN p.i.) from an *L. rhamnosus* or *L. brevis* colonization setting (see Materials and Methods). (C) LDH release at 24 h post-infection of IECs colonized by viable or inactivated (heat- or formaldehyde-treatment) *L. rhamnosus* or *L. brevis* (MOI 50) followed by infection with *C. albicans* (MOI 1). The results were normalized to *C. albicans* single infection. (D) LDH release at 24 h post-infection of IECs colonized by viable or inactivated (heat- or formaldehyde-treatment) *L. rhamnosus* or *L. brevis* (MOI 50) followed by infection with *C. albicans* (MOI 1) or added simultaneously at various MOI (50, 250, 500) with *C. albicans* infection (MOI 1). Single infections with *C. albicans* or lactobacilli were performed as controls. Results were normalized to *C. albicans* single infection. Data are mean±s.e.m. *P<0.05, **P<0.01, ***P<0.005 (*t*-test).

To determine whether damage protection was mediated by secreted bacterial compounds (James et al., 2016; Parolin et al., 2015; Santos et al., 2018; Tan et al., 2017; Wang et al., 2017), we analyzed the ability of *L. rhamnosus* to counteract *C. albicans-*induced cytotoxicity in a contact-independent manner. Supernatants (SN) from *Lactobacillus-*colonized gut models (Pre-Inc. SN) were unable to reduce *Candida-*mediated epithelial damage, whereas supernatants from *Lactobacillus* grown in MRS broth (MRS-SN) resulted in a trend of reduced damage (Fig. 5A). Supernatants obtained after *Lactobacillus* colonization or after 24 h of infection also did not influence hyphal length (Fig. 5B).

### Full damage protection requires viable lactobacilli

As high numbers of bacteria mediated protection against *C. albicans*-induced damage (Fig. 2A), we investigated whether *L. rhamnosus* needs to be alive and metabolically active or whether just sheer biomass and physical presence are sufficient to exert its protective effect. Neither heat- nor formaldehyde-killed lactobacilli were able to reduce *Candida*-mediated damage (Fig. 5C). As killed bacteria do not multiply on host cells, they may not reach the required biomass. Therefore, simultaneous infections of *C. albicans* with increasing numbers of killed lactobacilli were performed (Fig. 5D). Only extremely high numbers [multiplicity of infection (MOI) 500] of killed *L. rhamnosus* demonstrated a trend towards damage protection. However, even *L. brevis*, which did not mediate damage protection in other assays, induced a similar damage reduction at such high numbers. This indicates that the protection observed at these concentrations is likely due to different mechanisms from those of viable bacteria at lower numbers. Supporting this, viable *L. rhamnosus* cells at the same inoculum achieved almost 100% damage protection.

### Exopolysaccharides of *L. rhamnosus* are not involved in damage protection

Protection against *C. albicans* pathogenicity by lactobacilli was previously attributed to the presence of exopolysaccharides of the outer carbohydrate layer of *L. rhamnosus* GG (Allonsius et al., 2017). However, exopolysaccharide-deficient *L. rhamnosus* inhibited *C. albicans*-induced cytotoxicity to the same extent as wild-type *L. rhamnosus* (Fig. S2).

### Glucose consumption and lactate production are not responsible for *Lactobacillus*-mediated damage protection

Previous studies suggested that glucose consumption and lactate production by lactobacilli may be critical for the reduction of fungal damage potential (Hasslöf et al., 2010; Hütt et al., 2016; Köhler et al., 2012; Mailänder-Sánchez et al., 2017).

**Fig. 6.**
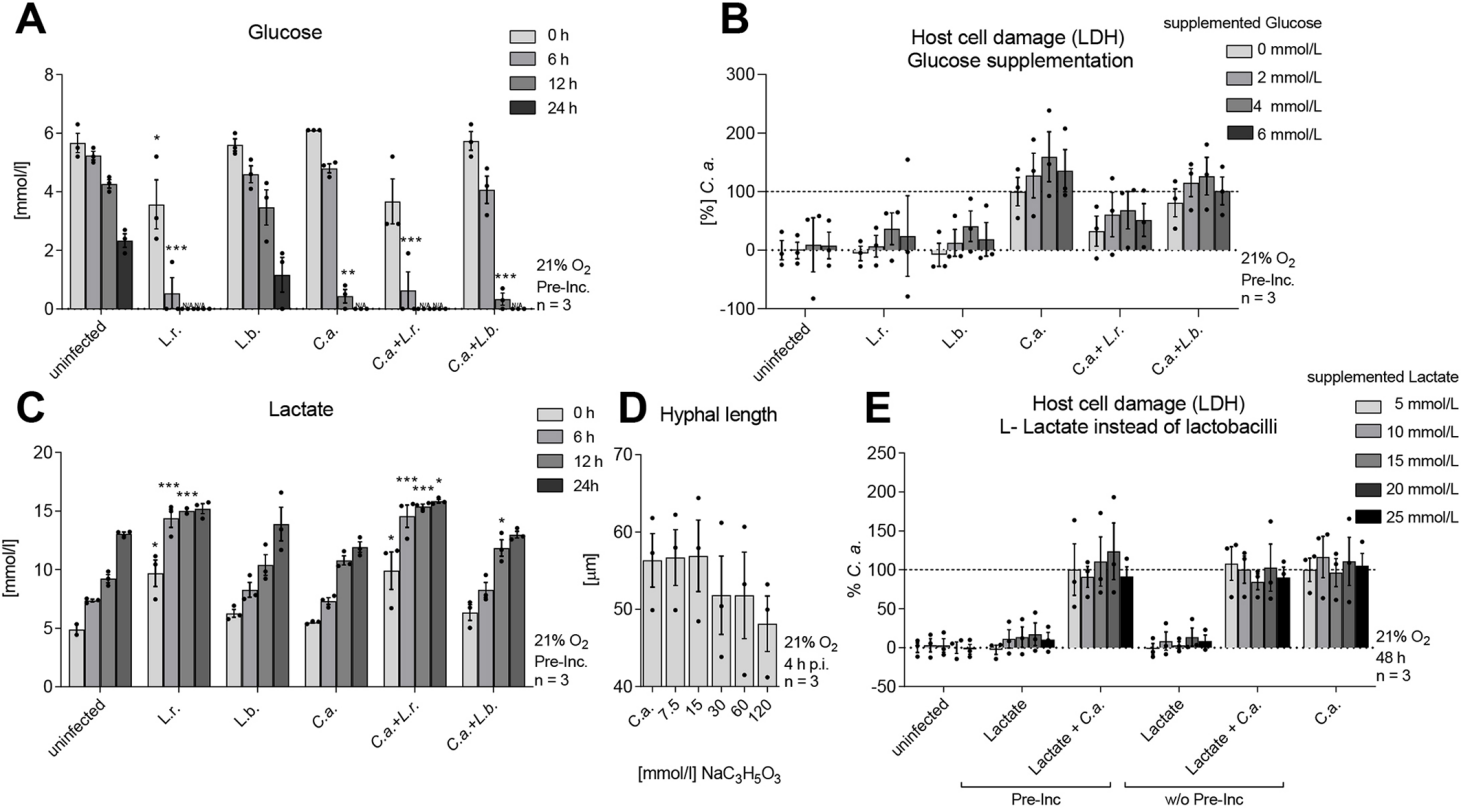
**Reduced glucose and increased lactate levels do not mediate *Lactobacillus*-driven damage protection.** (A,C) IECs were colonized with *L. rhamnosus* (L.r.) or *L. brevis* (L.b.) (MOI 50) or left uncolonized. Subsequently, cells were challenged or not with *C. albicans* (C.a.) (MOI 1). The amount of glucose (A) and lactate (C) was evaluated after colonization (0 h) and at 6, 12 and 24 h post-infection. (B) LDH release at 24 h post-infection of IECs colonized with *L. rhamnosus* and *L. brevis* and glucose supplementation (0-6 mmol/l) simultaneous to *C. albicans* (MOI 1) infection. (D) The effect of increasing concentrations of sodium L-lactate (0-120 mmol/l) on the hyphal length of *C. albicans* was measured microscopically after 4 h of incubation in KBM cell culture medium. (E) LDH release at 48 h post-infection of IECs either colonized or co-incubated with lactate (5-20 mmol/l) and infected with *C. albicans* (MOI 1) or not. The results were normalized to *C. albicans* single infection. Data are mean±s.e.m. *P<0.05, **P<0.01, ***P<0.005 (one-way ANOVA).

Although glucose levels dropped slowly when the model remained uninfected or was colonized with *L. brevis*, glucose was consumed within 12 h during *C. albicans* infection (Fig. 6A). Colonization with *L. rhamnosus* (in the presence or absence of *C. albicans* infection) already led to increased glucose consumption within the first 6 h of the experiment (Fig. 6A). As rapid glucose consumption is a mechanism by which *C. albicans* can cause damage to macrophages (Tucey et al., 2018), we speculated that the reduced glucose levels caused by *L. rhamnosus* might affect the potential of *C. albicans* to cause damage. However, when glucose was supplemented after colonization with lactobacilli, to compensate for the reduced glucose levels, no effect on *L. rhamnosus*-mediated damage protection was observed (Fig. 6B).

Lactate levels slowly increased in all conditions, but this effect was significantly enhanced by colonization with *L. rhamnosus* (Fig. 6C). As lactate was previously described to be a key mediator of *Lactobacillus* protection (Buffo et al., 1984; Hasslöf et al., 2010; Hütt et al., 2016; Köhler et al., 2012; Maudsdotter et al., 2011; Noverr and Huffnagle, 2004), we validated whether lactate itself affected filamentation of *C. albicans* in the medium used in our model. The influence of lactate on the extracellular pH was excluded in our model by buffering the cell culture medium, an essential requirement for the fitness of the intestinal cells. Increasing lactate concentrations in this setting did not affect hyphal length (Fig. 6D). In addition, lactate supplementation did not induce damage protection (Fig. 6E).

### *Lactobacillus*-mediated damage protection involves the shedding of *C. albicans*

**Fig. 7.**
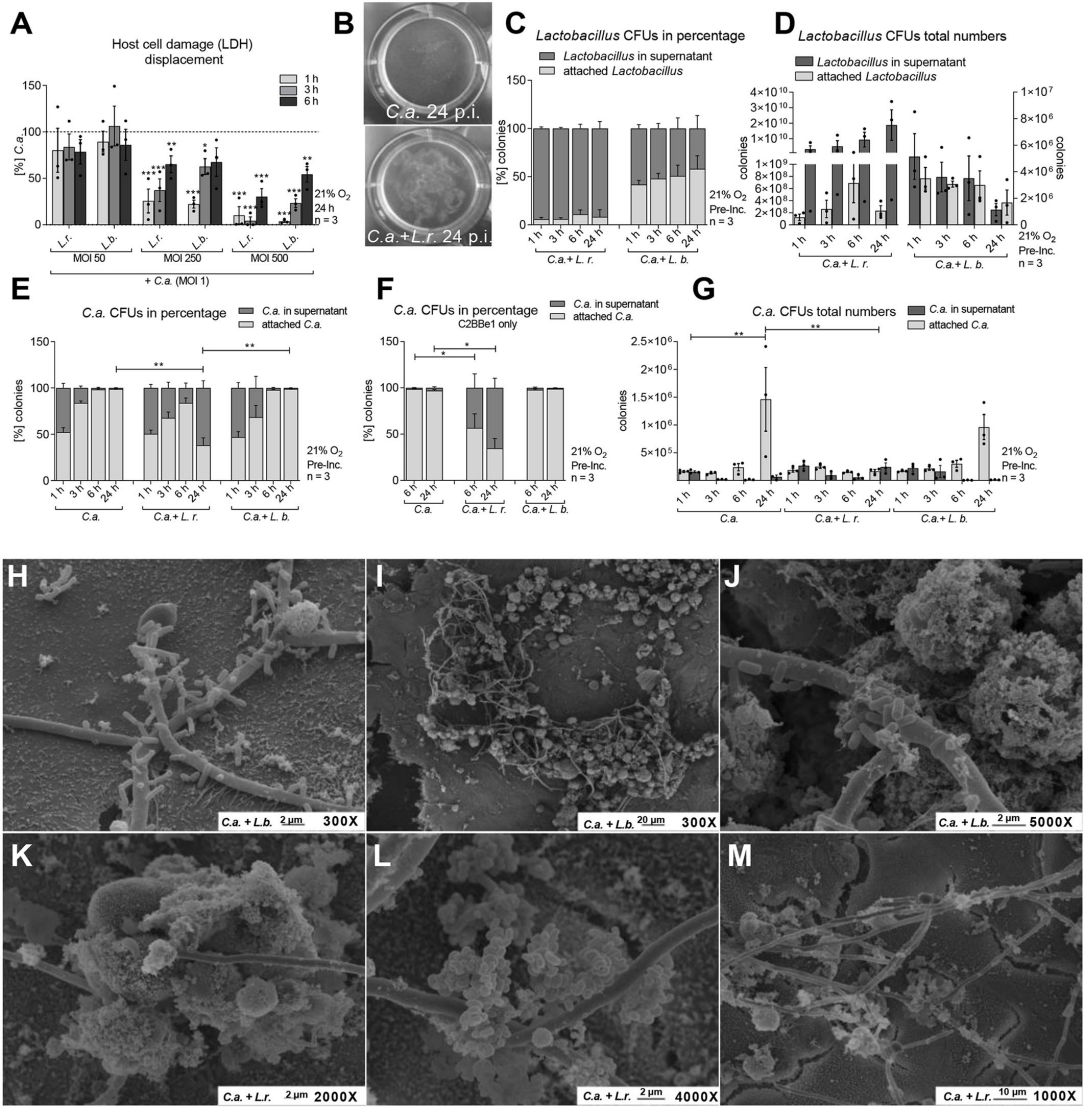
**Lactobacilli induce shedding of *C. albicans* and host cells.** (A) LDH at 24 h post-infection of IECs infected with *C. albicans* (C.a.) (MOI 1) for 1, 3 or 6 h and subsequently colonized with *L. rhamnosus* (L.r.) or *L. brevis* (L.b.) at various MOI (50, 250, 500). The results were normalized to *C. albicans* single infection. (B) Macroscopic observation of shedding at 24 h post-infection in a model of IECs colonized with *L. rhamnosus* or not and infected with *C. albicans* (MOI 1). (C,D) *L. rhamnosus* or *L. brevis* supernatant and cell-associated CFUs measured during the course of *C. albicans* infection at 1, 3, 6 and 24 h; data shown as relative percentages (C) or absolute numbers (D). (E-G) *C. albicans* supernatant and cell-associated CFUs measured during the course of *C. albicans* infection in untreated and *L. rhamnosus-* or *L. brevis-*colonized (MOI 50) IECs at 1, 3, 6 and 24 h (E,G) or 6 and 24 h post-infection (F); data shown as relative percentages (E,F) or absolute numbers (G). Data are mean±s.e.m. *P<0.05, **P<0.01, ***P<0.005 (A: *t*-test; D-G: one-way ANOVA). (H-M) Scanning electron micrographs of IECs colonized with *L. brevis* (H-J) or *L. rhamnosus* (MOI 50) (K-M) and infected with *C. albicans* for 6 h.

In order to invade and damage epithelial cells, *C. albicans* requires physical contact with the cells (Dalle et al., 2010; Wächtler et al., 2011). As we did not observe an influence of lactobacilli on adhesion of *C. albicans*, we speculated that damage protection could involve displacement of already bound fungal cells. Therefore, we performed displacement assays in which *C. albicans* was used to infect the model and, subsequently, viable lactobacilli were added 1, 3 or 6 h post-infection (Fig. 7A). The more bacterial cells were added, the stronger the damage was reduced. In addition, the longer the inoculation of lactobacilli was delayed, the less protection was achieved. Still, *L. rhamnosus* added 6 h after *C. albicans* infection reduced host cell damage by 60% at the highest inoculum, compared to 90% reduction when added 1 h post*-*infection.

An accumulation of debris was observed in the supernatant at later stages of infection, with more macroscopically observable debris when the model was colonized by *L. rhamnosus* (Fig. 7B). Thus, we quantified and analyzed the localization of lactobacilli and *C. albicans* cells during infection (Fig. 7C-G). The most substantial fraction of *L. rhamnosus* colony forming units (CFUs) was retrieved from the culture supernatant (Fig. 7C), and 1 h post-infection the number of CFUs doubled compared to the starting inoculum. Nevertheless, *L. rhamnosus* CFUs continued to increase drastically during *C. albicans* infection to almost 200-fold the initial inoculum (Fig. 7D). *L. brevis* appeared to be more localized at the epithelial barrier (Fig. 7C) and did not proliferate during the course of infection (Fig. 7D).

On the fungal side, independent of lactobacilli colonization, approximately half of the fungi were present in the supernatant 1 h post-infection, whereas the other half were localized within or attached to the epithelial barrier (Fig. 7E). During the course of infection, increasing percentages of fungi were associated with the gut epithelium layer over time, with 99% of fungal cells attached to host cells after 24 h (Fig. 7E). Colonization of the model with *L. rhamnosus* caused slightly decreased fungal association with the gut epithelial barrier over time, but 24 h post-infection the majority of fungal cells (62%) were found to be in the supernatant (Fig. 7E). Similar numbers were observed with gut models consisting of only C2BBe1 cells. Thus, this shedding was independent of the presence of HT29-MTX mucus-secreting goblet cells (Fig. 7F). In line with this, *L. rhamnosus* colonization also did not significantly influence gene expression or protein levels of mucin genes known to be expressed by intestinal tissue (Fig. S3). In terms of absolute numbers, *C. albicans* CFUs significantly increased at 24 h post-infection, yet colonization with *L. rhamnosus* also prevented *C. albicans* outgrowth and kept the number of viable fungi constant during the course of infection (Fig. 7G).

### Lactobacilli induce aggregation of *C. albicans* hyphae, bacteria and host cells

To investigate the cell-cell interactions in our model, we visualized the aggregation potential of microbial species to either themselves (auto-aggregation) or each other (co-aggregation) using scanning electron microscopy. Aggregation of both *Lactobacillus* species with *C. albicans* was observed (Fig. 7H-M). Six hours after infection of colonized IECs, almost all *C. albicans* hyphae were covered with *L. brevis* cells (Fig. 7H). Interestingly, bacterial cells were only found in contact with fungal cells; they did not adhere to host cells. *L. rhamnosus* formed large masses via auto-aggregation, which also stuck to *C. albicans* hyphae (Fig. 7K-M) and were rarely found on host cell layers.

### Shedding of *C. albicans* is associated with host cell apoptosis

**Fig. 8.**
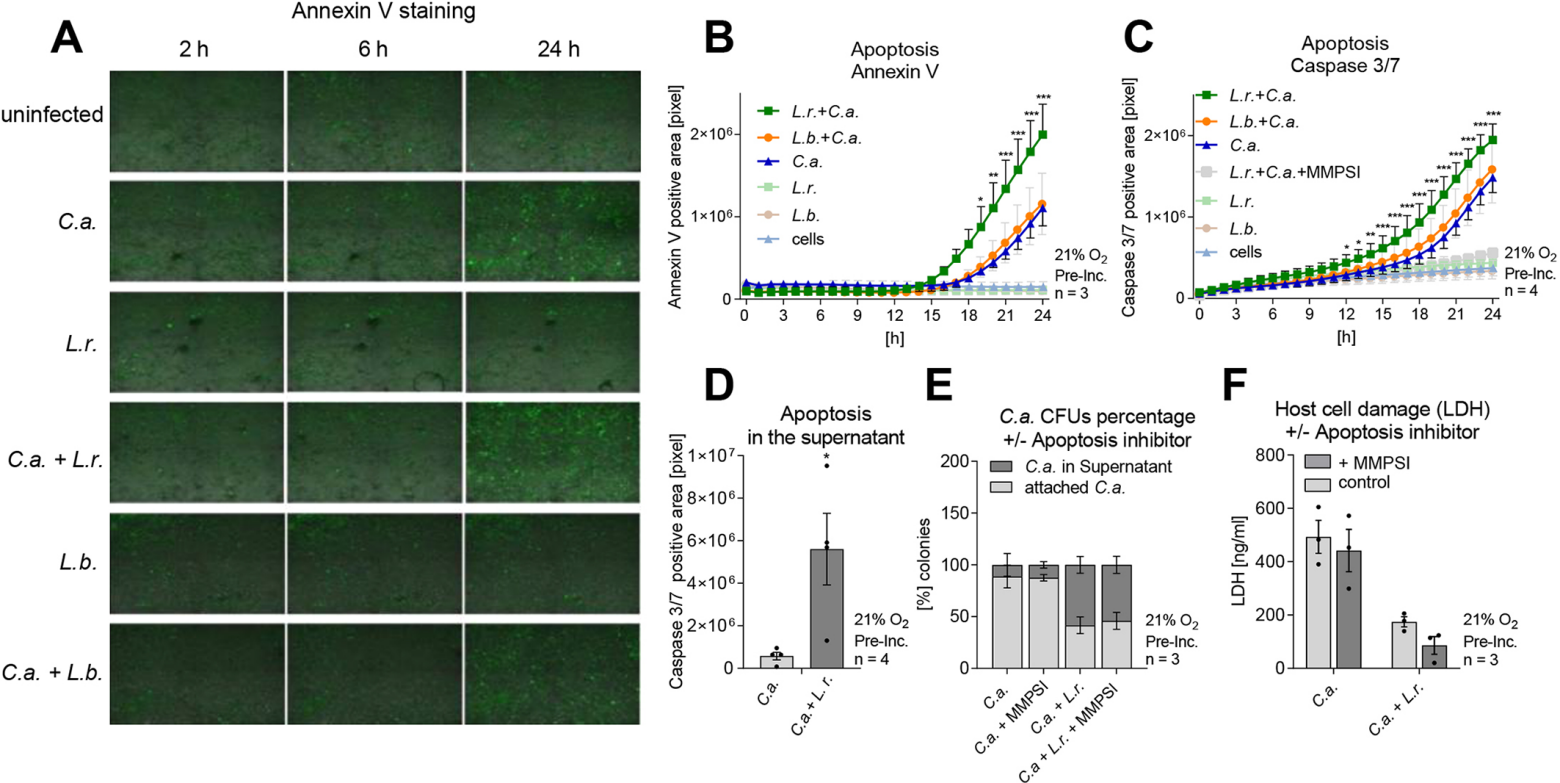
***Lactobacillus*-induced shedding of *C. albicans* correlates with host cell apoptosis.** (A-C) Apoptosis of IECs tracked during the course of *C. albicans* (C.a.) infection by live-cell imaging of annexin V expression (A,B) and caspase 3/7 activity (C). Apoptosis was followed for uninfected cells, *L. rhamnosus*- and *L. brevis*-colonized cells as well as *C. albicans*-infected cells in the presence and absence of *L. rhamnosus* (L.r.) or *L. brevis* (L.b.) colonization. Calculated area of annexin V-expressing cells (B) or cells with caspase 3/7 activity (C). (D) Caspase 3/7 activity in shed material at 24 h post *C. albicans* infection of IECs that were colonized with *L. rhamnosus* or left uncolonized. (E) relative percentages of *C. albicans* supernatant and cell-associated CFUs measured during the course of *C. albicans* infection in untreated and *L. rhamnosus-*colonized (MOI 50) IECs in the presence or absence of a caspase 3/7 inhibitor (MMPSI, 50 µM) at 24 h post-infection. (F) LDH release at 24 h post-infection of IECs colonized with *L. rhamnosus* or not infected with *C. albicans* (MOI 1) in the presence or absence of a caspase 3/7 inhibitor (MMPSI, 50 µM). Data are mean±s.e.m. *P<0.05, **P<0.01, ***P<0.005 (B,C: two-way ANOVA; D: *t*-test).

To maintain tissue homeostasis, the intestinal epithelium induces apoptosis and shedding of senescent cells, while the renewal of cells conserves barrier integrity. As we observed that *C. albicans* is shed in association with apoptotic host cells, live-cell imaging was used to investigate the influence of *L. rhamnosus* on host cell apoptosis as determined by extracellular phosphatidylserine exposure stained with annexin-V (ANXA5; Fig. 8A,B) and caspase 3/7 (CASP3/7) activity (Fig. 8C). Although *L. rhamnosus* and *L. brevis* alone did not induce host cell apoptosis (Fig. 8A-C), *C. albicans* infection did induce host cell apoptosis 16 h post-infection to some degree; however, colonization with *L. rhamnosus* significantly increased this induction (Fig. 8A-C). With *L. brevis*, the induction of apoptosis was similar to that of *C. albicans* alone (Fig. 8C). To verify that apoptotic cells were localized within the shed material, caspase 3/7 activity of epithelial cells within this material was quantified. When *L. rhamnosus* was used to colonize the model, significantly more epithelial cells with caspase 3/7 activity were observed in the shed material (Fig. 8D). To elucidate whether increased apoptosis in the presence of *L. rhamnosus* was a prerequisite for shedding, apoptosis was inhibited. Despite efficient inhibition of apoptosis through a selective caspase 3/7 inhibitor, no inhibition of shedding (Fig. 8E) or restoration of *C. albicans*-induced damage was observed (Fig. 8F).

## DISCUSSION

Epithelial cells exposed to *C. albicans in vitro* are rapidly and severely damaged indicating a high pathogenic potential in this *in vitro* context (Allert et al., 2018; Dalle et al., 2010; Wächtler et al., 2011). Here, we established a novel *in vitro* gut model in which we systematically explored the mechanisms that can prevent pathogenicity and help to achieve a commensal state. The inclusion of mucus-producing goblet cells reduced the adhesion of *C. albicans* to the epithelial barrier and its translocation, but not damage or hyphal length. To achieve protection against damage, a protective artificial ‘microbiota’ in the form of *Lactobacillus s*pecies with *Candida*-antagonizing potential needed to be co-cultured in the model. The presence of these live bacteria prevented *C. albicans* overgrowth and caused shedding of *C. albicans* from the host cells in a contact-dependent manner, which temporally correlated with increased host cell apoptosis. However, apoptosis was not required for *Lactobacillus*-induced shedding and protection against *C. albicans-*mediated damage. Although less potent than the presence of a protective microbiota, hypoxia also reduced *C. albicans-*mediated damage. Still, the antagonistic potential of *L. rhamnosus* was not augmented by hypoxic conditions. In this three-partite gut model of intestinal epithelial cells, a bacterial member of the gut microbiota and *C. albicans*, the pathogenic potential of *C. albicans* could be reduced.

Current *in vitro* models used to study *C. albicans* pathogenicity mostly rely on a monoculture of epithelial cells (Allert et al., 2018; Donnarumma et al., 2014; Mailänder-Sánchez et al., 2017; Rizzo et al., 2013). Here, a mixture of enterocytes and mucus-producing goblet cells was used to more closely mimic the intestinal epithelium (Beduneau et al., 2014; Ferraretto et al., 2018). Using this setup, adhesion and translocation of *C. albicans* were decreased, presumably owing to the presence of mucus. Nevertheless, epithelial damage as well as hyphal length remained unchanged. Therefore, an additional level of complexity was introduced in the form of an artificial microbiota. As microbiota, *Lactobacillus* species were selected for several reasons. First, they are found throughout the gastrointestinal tract of healthy humans (Finegold et al., 1977). Second, they are not pathogens themselves or fast-growing and, therefore, do not pose a threat to the epithelial cells. Third, they are widely described to have antagonistic properties against *C. albicans* (reviewed by Matsubara et al., 2016a and Förster et al., 2016). Fourth, *Lactobacillus* species are the most widely used probiotics to prevent or treat *Candida*-infections in clinical trials. *C. albicans* colonization of preterm neonates treated with probiotic *L. rhamnosus* was reduced (Manzoni et al., 2006). Furthermore, *L. reuteri* colonization was as effective as antifungal Nystatin prophylaxis in this patient group (Oncel et al., 2015) and can reduce late-onset sepsis (Romeo et al., 2011). A mixture of different *Lactobacillus* spp. strains, *Bifidobacterium* spp., and *Saccharomyces* spp. reduced gastrointestinal *C. albicans* colonization as well as candiduria as a result of disseminated infections in critically ill children receiving broad-spectrum antibiotics (Kumar et al., 2013). Of note, clinical data are limited on the use of probiotics in immunocompromised patients treated with broad-spectrum antibiotics, the major risk group for systemic candidiasis. Yet, despite the extensive evidence that lactobacilli antagonize *C. albicans*, direct interactions on mucosal surfaces mimicking the gastrointestinal tract have not been described.

Out of six tested *Lactobacillus* species, *L. rhamnosus*, *L. paracasei*, *L. casei* and *L. salivarius* were capable of preventing *C. albicans*-induced cytotoxicity. Antifungal activity for these *Lactobacillus* species was also described in Allonsius et al. (2019). For a more in-depth analysis of the reduced damage, protective *L. rhamnosus* was compared with the less protective species *L. brevis*. The reduced damage following *L. rhamnosus* colonization correlated with reduced hyphal elongation, fungal translocation and shedding of fungal filaments in conglomerates consisting of apoptotic host cells, hyphae and bacteria. Reduced induction of damage- and stress-related host responses (MAPK and NF-κB signaling), which were previously shown to be part of the damage response to *C. albicans* in epithelial cells (Moyes et al., 2010), validated the improved condition of the epithelial barrier on a molecular level.

Many studies demonstrated that *Lactobacillus* supernatants restrict growth, yeast-to-hyphal-transition and biofilm formation (James et al., 2016; Parolin et al., 2015; Santos et al., 2018; Tan et al., 2017; Wang et al., 2017). In contrast, we observed that damage protection exerted by *L. rhamnosus* was contact dependent. Of note, these studies used a variety of *Lactobacillus* strains, and the production of antimicrobial metabolites is highly strain specific. In agreement with Felten et al. (1999), who showed that *L. rhamnosus* is not an H_2_O_2_ producer, no hydrogen peroxide production was detected for any of the *Lactobacillus* species tested in our study (data not shown). Likewise, the production of biosurfactants and bacteriocins, which are anti-candidal and decrease adhesion and biofilm formation (Fracchia et al., 2010; Hütt et al., 2016; Kaewsrichan et al., 2006; Morais et al., 2017; Parolin et al., 2015; Strus et al., 2005; Zakaria Gomaa, 2013), played only a minor role, if any, in our study, as *Lactobacillus* supernatants did not reduce damage. Even though colonization of *L. rhamnosus* resulted in lactate accumulation, its supplementation at concentrations comparable to those in *L. rhamnosus*-colonized models did not inhibit *C. albicans*’ cytotoxicity or hyphal elongation. This can likely be attributed to the buffered KBM cell culture medium (required to keep the epithelial cells healthy in our model), which kept the pH neutral throughout the experiment and thus dampened any potential antimicrobial effect of lactic acid shown in other studies at acidic pH (Buffo et al., 1984; Hasslöf et al., 2010; Hütt et al., 2016; Köhler et al., 2012; Maudsdotter et al., 2011). Nevertheless, acidification via lactate production may contribute to protection against *C. albicans* pathogenicity *in vivo*. However, our data suggest the necessity of physical interaction between *L. rhamnosus* and *C. albicans* for damage protection in the setting of our gut model.

A crucial aspect of our study is the observation that live *L. rhamnosus* cells are required for reduced *C. albicans* pathogenicity. Nevertheless, an extreme biomass of killed bacteria, even the non-protective *L. brevis* strain, mediated protection. The four protective *Lactobacillus* species (*L. rhamnosus*, *L. paracasei*, *L. casei* and *L. salivarius*) proliferated on host cells. Thus, overnight colonization of a low bacterial cell number yielded the required protective biomass. As *L. brevis* and *L. fermentum* did not grow under the given conditions, the two species are only protective at higher inocula, as shown in simultaneous infections and displacement experiments. This means that the ability to replicate during colonization before infection with *C. albicans* distinguishes the protective from less-protective *Lactobacillus* species. In other words, the reduced growth of *L. brevis* and *L. fermentum* in our model appears to be the key reason why these species have a limited protective potential, as they otherwise have similar attributes to the protective species. Interestingly, the growth of lactobacilli required the presence of host cells, as growth in KBM medium alone was not observed for any species. Adhesion to mucin-coated surfaces can shift lactobacilli to a more protease-active phenotype, thereby degrading mucin as a nutrient source for the bacteria (Wickström et al., 2013). Another possibility is the formation of hypoxic microniches when intestinal cells are present, resulting in the growth of microaerophilic bacteria that can bear oxygen, but prefer environments containing lower levels of oxygen for growth. *L. rhamnosus* even showed increased proliferation during *C. albicans* infection, which could suggest that *C. albicans* may additionally support *Lactobacillus* growth. A potential explanation could be hypoxic microniches that are formed by metabolically active *C. albicans* hyphae (Lambooij et al., 2017). The *Lactobacillus* species used in the study differ in their ability to utilize environmental conditions for replication. Reaching a critical biomass of ∼200 bacteria per *C. albicans* cell was indispensable for *Lactobacillus*-mediated inhibition of *C. albicans*. Our data show a significant expansion of the *L. rhamnosus* population during colonization and over the course of infection, which easily reaches this biomass of viable bacteria. Nevertheless, killed *L. rhamnosus*, and even *L. brevis*, reduced fungal-induced damage to some extent, but not in a manner akin to viable bacteria. It is likely that steric hindrance influences the cytotoxicity of *C. albicans*. Incidentally, this was also observed when using killed *C. albicans* cells instead of killed bacteria (Fig. S4). Still, for potent damage protection, viable lactobacilli were essential.

It can only be speculated whether a ratio of 200:1 can be reached in patients treated with *L. rhamnosus* probiotics. Clearly, colonization of the gastrointestinal tract in the stomach, ileum and colon (Alander et al., 1997, 1999; Valeur et al., 2004) can be achieved. However, the extent of colonization by lactobacilli varies drastically between studies with various formulations, treatment regimens and colonization readouts (Alander et al., 1999; Piano et al., 2012; Valeur et al., 2004). Nevertheless, the studies mentioned above show that the current probiotic treatment strategies are efficient in pre-term neonates, though knowledge is lacking regarding the exact quantitative interactions in such clinical settings, as well as the effect in an adult patient population.

Scanning electron microscopy revealed the ability of *L. rhamnosus* to co-aggregate with fungal cells. Therefore, the bacteria may reside in microniches that enable close contact cell-cell interactions. Within such an interface, *L. rhamnosus* may form a hostile microenvironment that mediates anti-*Candida* activities. Antimicrobial factors (mentioned above) were likely produced by the lactobacilli in our study, but were ineffective in the supernatant, probably because such factors did not reach minimum inhibitory concentrations. However, the same factor may inhibit *C. albicans* when higher concentrations are achieved in microniches. The concentration of short-chain fatty acids and other metabolites may be even higher, whereas nutrient availability may be even lower within these niches. Competition for nutrients could be one explanation. In agreement with Mailänder-Sánchez et al. (2017), damage prevention in the presence of *L. rhamnosus* was accompanied by glucose depletion*.* Despite the deprivation of the primary carbon source of *C. albicans*, supplementation of glucose at levels similar to those amounts consumed by *L. rhamnosus* during the colonization phase did not restore *C. albicans*’ damage potential. However, we cannot exclude that nutrient competition in microniches within the bacteria–*Candida* interface may contribute to growth inhibition and shedding of *C. albicans*.

Several studies indicate an association between co-aggregation of protective bacteria with pathogens with the interference of adhesion to host cells. This involves bacterial surface molecules such as peptidoglycan, teichoic acids, (glyco)proteins and polysaccharides (Allonsius et al., 2017; Coman et al., 2015; Kang et al., 2018; Kleerebezem et al., 2010; Lebeer et al., 2012, 2010; Malik et al., 2016; Niu et al., 2017; Parolin et al., 2015; Santos et al., 2016; Zivkovic et al., 2015). In the case of IECs, we observed striking differences to data obtained in many other studies. For example, exopolysaccharides of *L. rhamnosus* GG were shown to interfere with hyphal formation and *C. albicans* adhesion to vaginal epithelial cells (Allonsius et al., 2017; Donnarumma et al., 2014). However, the presence of lactobacilli in our model did not influence the binding efficiency of the fungus. Accordingly, an exopolysaccharide-deficient *L. rhamnosus* GG mutant (Allonsius et al., 2017) conveyed protection to a similar extent as the wild type in our study. The fact that *C. albicans* adhesion remained unaffected in our model and the finding that exopolysaccharides were not involved in damage reduction suggests mechanisms different from those shown in other studies.

Filamentation is one of the most essential virulence attributes of *C. albicans* through which the fungus can invade tissue and is associated with cytotoxicity (Dalle et al., 2010; Gow et al., 2011; Höfs et al., 2016; Moyes et al., 2016). Lactobacilli-influenced hyphal length is potentially supported by the mucus-producing goblet cells (Allonsius et al., 2019; Kavanaugh et al., 2014) and likely contributes, in part, to the reduced cytotoxicity. However, we propose that the actual mode of action of *Lactobacillus*-mediated damage protection is likely inhibition of fungal overgrowth and shedding of fungal cells, which already takes place after 6 h of infection. After 24 h, the majority of *Candida* cells were located in the supernatants, spatially restricting fungal cells from host cells and preventing invasion and hyphal-associated damage of the epithelial cell layer. Indeed, the presence of *L. rhamnosus* reduced *C. albicans* translocation. To our knowledge, inhibition of adhesion was mainly studied on vaginal and oral epithelial cells (Allonsius et al., 2017; Mailänder-Sánchez et al., 2017). This means that beneficial effects of lactobacilli, which are often considered species- and even strain-specific, likely also depend on the epithelial environment within the human body.

*Lactobacillus*-induced mucus production correlates with the inhibition of attachment of various pathogens to epithelial surfaces (Hafez, 2012; Kim et al., 2008; Li et al., 2008; Zivkovic et al., 2015). This was also considered as a mechanism that could have contributed to the detachment and shedding of *Candida* hyphae. However, no influence of *L. rhamnosus* on mucin gene or protein expression was observed and it can, therefore, be assumed that an increased mucus production has little, if any, contribution to the detachment of *C. albicans* from *Lactobacillus*-colonized intestinal epithelial cells. Consistent with this, shedding was also induced in the absence of mucus-secreting goblet cells.

Besides protective mucus, the intestinal epithelium is known for its very high turnover rate. Enterocytes have a short lifetime and are continuously shed into the lumen of the gut, completely renewing the epithelium every 5-7 days. In humans, this daily loss has been estimated at 10^11^ cells (Potten, 1990). Fully differentiated intestinal epithelial cells experience detachment-induced, caspase-dependent apoptosis (a programmed cell death termed ‘anoikis’) (Beauséjour et al., 2012; Bullen et al., 2006; Patterson and Watson, 2017). This process is accompanied by perturbations of tight junctions, which are rapidly reassembled by neighboring cells to maintain barrier integrity (Guan et al., 2011; Williams et al., 2015). We observed that the shed *Candida*-bacteria aggregates contained host cells with apoptotic characteristics such as phosphatidylserine expression and caspase 3/7 activity. Furthermore, we observed that *L. rhamnosus* increased expression of apoptotic markers of epithelial cells, without increasing LDH release. Apoptotic cells, in contrast to necrotic cells, retain their membrane integrity (Zhang et al., 2018). Therefore, the observation of apoptotic markers in the absence of LDH release points towards induction of apoptosis by *L. rhamnosus* during infection with *C. albicans*. In the literature, contrasting results were described concerning *Lactobacillus-*mediated apoptosis induction. Some studies demonstrate the ability to prevent pathogen-induced apoptosis (Li et al., 2008; Santos et al., 2016), whereas others state the opposite (Sungur et al., 2017; Zielinska et al., 2018). Interestingly, the timing of the apoptosis induction coincided with the observed shedding. Nevertheless, blockade of apoptosis via inhibition of caspase 3/7 did not reduce shedding nor did it restore *C. albicans*-induced damage in the presence of *L. rhamnosus*. This could suggest that apoptosis might be a consequence of shedding rather than the mechanism causing it. We therefore propose that the *L. rhamnosus-*induced shedding of *C. albicans* hyphae may result in detachment of epithelial cells to which the hyphae are attached, resulting in induction of apoptosis due to a loss of anchorage.

Although phosphatidylserine exposure and caspase 3/7 activity were also observed during infection with *C. albicans* alone, this was paired with increased LDH release, suggesting predominantly necrotic cell death (Allert et al., 2018; Chan et al., 2013). Furthermore, the epithelial integrity measured by TEER showed a decrease when exposed to *C. albicans* alone. Allert et al. (2018) stated that loss of epithelial integrity and *C. albicans-*induced damage can be independent processes – meaning for the current study that colonization with *L. rhamnosus* can lead to TEER loss (presumably by shedding of apoptotic cells and renewal of the epithelial barrier) and a simultaneous inhibition of *C. albicans* translocation*.* Nevertheless, it remains unknown why TEER is reduced upon colonization of *L. rhamnosus* alone, as no induction of apoptosis was observed under these conditions. This reduction of TEER in the presence of *L. rhamnosus* contrasts the other protective effects imposed upon *C. albicans* pathogenicity. This also contrasts with previous studies demonstrating that *Lactobacillus* species can even improve barrier integrity (Barnett et al., 2018).

Although the molecular mechanism behind the shedding observed in this study remains unknown, it appears to be clear that this is the result of a tripartite interaction of bacteria, *C. albicans* and epithelial cells rather than due to dual interactions between two of the players in this model. Whether shedding is an exclusive feature of *L. rhamnosus* or is also induced by other *Candida-*antagonizing bacteria, remains to be investigated. Similarly, shedding may also be a protective feature of other epithelial tissues colonized by *Candida-*antagonizing bacteria. Even though supernatants of *L. rhamnosus* cultured within the model were not capable of inhibiting *C. albicans* pathogenicity, yet undiscovered metabolic interactions may play a role in *Lactobacillus-*mediated damage protection.

We are convinced that our model, which is to our knowledge the first *in vitro* gut model to mimic a more commensal (non-damaging) state of *C. albicans*, can provide crucial insights to understanding the interplay between host cells and microbiota in preventing pathogenicity. The analysis of *C. albicans* as a commensal using *in vivo* models is hampered by the fact that most laboratory animals (including mice) are not natural hosts of *C. albicans* and colonization requires germ-free animals or an antibiotic-induced dysbiosis (Fan et al., 2015; Koh, 2013; Naglik et al., 2008). The model developed here could facilitate the study of *C. albicans* commensalism and pathogenesis with human cells. Increasing our current knowledge on the commensal state of *C. albicans* is crucial to understand how it gained its pathogenic potential (Hube, 2004) and to explain biological effects observed in *in vivo* clinical trials, which, for example, showed the usefulness of lactobacilli for the prevention and treatment of vulvovaginal and oral candidiasis (Hatakka et al., 2007; Kumar et al., 2013; Manzoni et al., 2011, 2006; Martinez et al., 2009). We believe that our experimental model and refined future modifications, e.g. containing immune cells, for *C. albicans* commensalism can be widely applied for future studies investigating the interaction between *C. albicans* and other members of the microbiota in a more biologically relevant context*.*

Collectively, we established a commensal-like model to study *C. albicans* interactions in the presence of antagonistic *Lactobacillus* species and intestinal epithelial cells including mucus-producing goblet cells (Fig. S1). Mechanistically, these bacteria induce shedding of invading *C. albicans* in aggregates with bacteria and host cells, thereby spatially restricting *C. albicans* from the epithelial barrier. This novel *in vitro* model may provide a stepping stone for more complex models aimed at studying the commensal and pathogenic states of *C. albicans* and could serve as a tool to study novel therapies aiming at preventing *C. albicans* pathogenicity.

## MATERIALS AND METHODS

### Microbial strains and culture conditions

The *C. albicans* wild-type strain SC5314 was grown on YPD plates (2% peptone, 1% yeast extract, 2% glucose, 2% agar) at 30°C. For use in experiments, *C. albicans* cells were grown overnight (o/n) in YPD medium (2% peptone, 1% yeast extract, 2% glucose) at 30°C and 180 rpm in a shaker incubator. Before infection, yeast cells from an o/n culture were collected by centrifugation at 20,000 ***g*** and washed twice with phosphate-buffered saline (PBS). The cell number was determined using a Neubauer chamber system, and the concentration adjusted to 4×10^5^ cells/ml in serum-free Keratinocyte Basal Medium (KBM, Lonza). For the killing of *C. albicans*, fungal cells were treated for 10 min at 70°C and the viability was proven by the absence of growth on YPD plates. *Lactobacillus* strains [*L. brevis*: ATCC, 14869; *L. casei*: ATCC, 393; *L. fermentum*: ATCC, 14931; *L. paracasei*: ATCC, 11578; *L. rhamnosus*: ATCC, 7469; *L. salivarius*: ATCC, 11741; *L. rhamnosus*: CMP5351 (Lebeer et al., 2009); *L. rhamnosus* GG: ATCC, 53103 (Lebeer et al., 2009)] were grown on Man, Rogosa and Sharpe (MRS) agar plates (Carl Roth) at 37°C and 1% O_2_. For use in experiments, bacterial cells were grown at 37°C without agitation in MRS broth (Carl Roth). Before experiments, lactobacilli were collected by centrifugation at 20,000 ***g***, washed twice in PBS and diluted to an optical density OD_600_ of 1 in KBM medium equaling a cell number of ∼1×10^8^ CFU/ml. For the killing of lactobacilli, bacterial cells were either treated for 30 min in Histofix (Carl Roth) or for 10 min at 90°C. Viability was proven by the absence of growth on MRS agar plates.

### Cell culture

The human intestinal epithelial Caco-2 subclone Caco2 brush border expressing 1 (C2BBe1; ATCC, CRL2102™; Peterson and Mooseker, 1992) and the human intestinal goblet cell HT29-MTX (ATCC, HTB-38; CLS, Lot No. 13B021) were routinely cultivated in Dulbecco's Modified Eagle's Medium (DMEM) (Gibco, Thermo Fisher Scientific) supplemented with 10% fetal calf serum (FCS) (Bio&Sell), 10 µg/ml Holotransferrin (Calbiochem, Merck) and 1% non-essential amino acids (Gibco, Thermo Fisher Scientific) at 37°C and 5% CO_2_ for no longer than 15 passages. Cell lines have been authenticated via commercial STR profiling (Eurofins Genomic) and checked for contaminations using a PCR mycoplasma test kit (PromoKine) according to the manufacturer's instructions. For detachment, C2BBe1 cells and HT29-MTX cells were treated with Accutase or 0.05% Trypsin-EDTA solution (Gibco, Thermo Fisher Scientific), respectively. For cell culture maintenance, cells were seeded in fresh 75 cm^2^ culture flasks containing supplemented DMEM at a 1:4 split ratio. For use in experiments, the cell numbers were determined using a Neubauer chamber system, and a mixture of C2BBe1:HT29-MTX cells at a 70:30 ratio was seeded in collagen I-coated [10 µg/ml collagen I for 2 h at room temperature (RT); Invitrogen, Thermo Fisher Scientific] well plates at an initial cell density of 2×10^4^ cells/well in a 96-well plate, 1×10^5^ cells/well in a 24-well plate and 4×10^5^ cells/well in a 6-well plate. IECs were cultured with regular medium exchange for 14 days post-seeding for differentiation before experimental use.

### Infection of IECs

For infection experiments, IECs were cultivated in serum-free KBM. Monolayers were either colonized (pre-incubated; pre-Inc.) with lactobacilli for 18 h before infection with *C. albicans*, or bacterial and fungal cells were added simultaneously (Fig. 1E). For displacement experiments, the sequence of inoculation was reversed. In this case, IECs were infected with *C. albicans* 1, 3 or 6 h before the addition of lactobacilli. Incubation was then continued for a total infection period of 24 h. For the exact number of cells (host, lactobacilli, and *C. albicans*) and incubation periods used in the respective experiments see Table S1. In addition to the coinfection of *C. albicans* and lactobacilli, single infections were carried out as controls.

### Quantification of cytotoxicity (LDH)

The influence of lactobacilli on *C. albicans*-mediated host cell damage was investigated by measuring the release of cytoplasmic LDH (Chan et al., 2013) as a proxy for loss of membrane integrity, a hallmark of necrosis (Zhang et al., 2018). LDH was quantified in the supernatant of infected IEC monolayers 24-48 h post-infection using the Cytotoxicity Detection Kit (Roche) according to the manufacturer's instructions. LDH from rabbit muscle (5 mg/ml, Roche) was used to generate a standard curve for the determination of LDH concentrations. The background control level of uninfected IECs was subtracted from the experimental LDH release and usually compared to 100% *C. albicans* single infection.

### Adhesion and filamentation of *C. albicans*

Upon colonization with lactobacilli, adhesion of *C. albicans* to epithelial cells was determined 1 h post-infection (for infective doses see Table S1). Non-attached *C. albicans* cells were removed by washing twice with PBS. Samples were fixed with Histofix for 15 min at RT or o/n at 4°C and subsequently rinsed three times with PBS. Adherent fungi were stained with Calcofluor White [10 µg/ml in 0.1 M Tris-HCl (pH 9.0), Sigma-Aldrich] for 20 min at RT in the dark. After washing three times with water, samples were mounted on glass slides with ProLong mounting medium (Thermo Fisher Scientific) and analyzed using fluorescence microscopy. The number of adherent *C. albicans* cells was determined in random fields of a defined size, allowing calculation of the adhesion percentage versus inoculated *C. albicans* cells.

Hyphal length of *C. albicans* was analyzed using differential staining according to Wächtler et al. (2011), with the following minor modifications. Briefly, after 4 h of *C. albicans* infection, IECs were washed three times with PBS and fixed with Histofix. Extracellular non-invasive fungal components were stained by incubation with a primary antibody against *C. albicans* (1:2000 in PBS, rabbit anti-*Candida*, BP1006, Acris Antibodies) for 1 h at 30°C, washing three times with PBS and incubating with a secondary antibody (1:5000 in PBS, goat anti-rabbit AlexaFluor488, A-11008, Thermo Fisher Scientific) for 1 h at 30°C. After rinsing three times with PBS, epithelial cells were permeabilized with 0.5% Triton X-100 (Sigma-Aldrich) for 10 min at RT and washed again three times with PBS. Entire *C. albicans* hyphae were stained with Calcofluor White [10 µg/ml in 0.1 M Tris-HCl (pH 9.0)] for 20 min at RT in the dark followed by washing three times with water. Stained samples were mounted on glass slides and visualized using fluorescence microscopy. The total hyphal length was also recorded for at least 100 hyphae per condition.

### Hyphal length influenced by sodium L-lactate

A *C. albicans* o/n culture was adjusted to 4×10^5^ cells/ml in KBM media. Sodium L-lactate (Sigma-Aldrich) was adjusted to a concentration of 0-120 mmol/l in a total volume of 375 µl per well in a 24-well plate (see Table S1). Cells were fixed with Histofix 4 h post-infection with *C. albicans*, and analyzed using a Cell Discoverer 7 (Carl Zeiss) with a 10× magnification. At least 100 hyphae per condition were measured to estimate average hyphal length.

### *In vitro* translocation

For measuring translocation, IECs were cultivated for 14 days on collagen I-coated transwell inserts with a 6.5 mm diameter and 5 µm pore size (Corning). Following colonization with lactobacilli, cells were infected with 2×10^4^ *C. albicans* cells per transwell for 24 h at 37°C and 5% CO_2_ (see Table S1). TEER values were measured using a voltmeter (World Precision Instruments) before and after colonization and 24 h post-infection. The resistance of a blank insert (120 Ω) was subtracted from each value. The translocation rate 24 h post-infection was measured using the following procedure. Zymolyase (260 U/ml in serum-free DMEM; Amsbio) was added to the lower compartment to a final concentration of 20 U/ml and incubated for 2 h at 37°C and 5% CO_2_. The detached *C. albicans* hyphae were then collected and plated at appropriate dilutions on YPD agar plates. Plates were incubated at 30°C for 1-2 days until adequate growth for determining colony-forming units (CFUs) was reached.

### Effects of lactobacilli cell-free supernatants

Lactobacilli were grown in MRS broth for 24 h at 37°C. The MRS-SN was prepared by centrifuging the culture at 4700 ***g*** for 5 min at 4°C, filtering through 0.22 µm filters (Millipore) and rebuffering against KBM medium within a 3K Amicon Ultra centrifugal filter device (Millipore) (final pH 7.3) according to the manufacturer's instructions. Moreover, IECs differentiated in 6-well plates were colonized with lactobacilli for 18 h. The culture supernatant (Pre-Inc. SN) was filtered through 0.22 µm filters (Millipore). Both supernatants were stored at 4°C until use in experiments the same day (see Table S1). Host cell damage induced by *C. albicans* infection in the presence of lactobacilli supernatants was measured 24 h post-infection by quantification of LDH (for format and volume see Table S1).

### Glucose and lactate measurements

Glucose and lactate were measured in supernatants of the *in vitro* model (KBM media) in a 6-well format (see Table S1). We collected 120 µl of the supernatant after 0, 6, 12 and 24 h and analyzed using the Abbott Architect ci8200 Integrated System (Abbott Laboratories) according to the manufacturer's protocol. Basal levels of the KBM medium were 9 mmol/l glucose and <0.17 mmol/l lactate.

### Quantification of shedding of *C. albicans* and lactobacilli – CFU determination

Shedding of *C. albicans* and lactobacilli was measured upon infection of *Lactobacillus*-colonized IECs for the respective time periods (see Table S1). Supernatants were collected and vortexed thoroughly. IECs were treated with 0.2% Triton-X-100 (Sigma-Aldrich) to lyse the host cells and release adherent fungal and bacterial cells. Supernatant and lysate samples were diluted appropriately with PBS. To follow the growth of *C. albicans*, the diluted samples were plated on YPD plates with 1× PenStrep (Gibco, Thermo Fisher Scientific) and incubated at 30°C for 1-2 days until adequate growth for determining the CFUs was reached. For lactobacilli, MRS plates with Nystatin (50 µg/ml; Carl Roth) were used for plating and incubated at 37°C and 1% O_2_ for 2 days.

### Scanning electron microscopy (SEM)

IECs differentiated for 14 days in a 24-well plate on glass coverslips were colonized with lactobacilli for 18 h and infected with *C. albicans* for 6 h (see Table S1). The SEM samples were fixed by incubating them in cacodylate-buffer containing 2.5% glutaraldehyde o/n at 4°C. Afterwards samples were washed three times in cacodylate buffer and dehydrated in an ethanol series (30%, 50%, 70%, 80%, 90%, 100%, 100% each 15 min), followed by critical point drying in a Leica EM CPD300 Automated Critical Point Dryer. The samples were sputter-coated with platin (layer thickness 20 nm) in a CCU-010 Compact Coating Unit (Safematic). Finally, samples were analyzed at different magnifications using a Zeiss (LEO) 1530 Gemini field emission scanning electron microscope (Carl Zeiss) at 7 kV acceleration voltage and a working distance of 5 mm using an InLens secondary electron detector.

### Isolation of human RNA

At the indicated time points uninfected, single infected and mixed infected IECs were harvested by removing supernatants and adding RLT buffer (Qiagen) to the host cell lawn. The cell lysates were further processed with the RNeasy mini kit (Qiagen) according to the manufacturer's protocol. Human RNA quantity was determined using a NanoDrop 1000 Spectrophotometer (Thermo Fisher Scientific) and RNA quality was verified with a 2100 Bioanalyzer (Agilent Technologies).

### Reverse transcription quantitative real-time PCR (RT-qPCR)

We reverse transcribed 500 ng of high-quality DNase I-treated RNA samples into cDNA using oligo-dT primers and SuperScript™ III Reverse Transcriptase (Life Technologies). Subsequently, 1 µl of diluted cDNA was used for gene expression analyses with EVAGreen^®^ qPCR Mix (Bio&Sell) and a C1000 thermocycler (Bio-Rad). Expression levels of biological triplicates were normalized to the reference genes *ACT1* and *GAPDH*. Primers used for qPCR analyses are listed in Table S2.

### Mucin ELISAs

Differentiated IECs were colonized with lactobacilli and infected with *C. albicans* for 24 h (see Table S1). For the measurement of released and membrane-bound mucin proteins, supernatant samples were collected and IECs were lysed by treatment with 75 µl RIPA-buffer (Millipore) containing protease inhibitors (cOmplete Protease Inhibitor Cocktail, Roche). Supernatant and lysate samples were mixed and centrifuged at 1000 ***g*** for 10 min at 4°C. Mucin concentrations were quantified using enzyme-linked immunosorbent assay (ELISA) according to the manufacturer's instructions (Human MUC 2, 3, 4, 13 and 17, ELISA Kit, DlDevelop; Human MUC5AC, ELISA Kit, Elabscience).

### Apoptosis

Differentiated IECs were colonized with lactobacilli or not and infected with *C. albicans* or not (see Table S1). Simultaneously with *C. albicans* infection, IECs were stained for phosphatidyl serine expression using annexin V (pSIVA™ Real-Time Apoptosis Kit; Bio-Rad) and caspase 3/7 activity (CellEvent™ Caspase 3/7 detection reagent; Invitrogen, Life Technologies) according to the manufacturer's instructions. Apoptosis was followed by live-cell imaging of the fluorescence at an excitation maximum of 488 nm (annexin V or caspase 3/7) every 1 h using a Cell Discoverer 7 (Carl Zeiss) with a 10× magnification. Images were processed using the Fiji software (ImageJ). After conversion to binary images, the total area of positive cells was determined using the Particle Analyzer tool. To inhibit apoptosis, the caspase 3/7 inhibitor 1 MMPSI (Abcam) dissolved in ethanol with a final concentration of 50 µM was used.

### Quantification of shedding of apoptotic cells

Differentiated IECs were colonized with lactobacilli or not and infected with *C. albicans* or not (see Table S1). Supernatants were collected, placed in a new 24-well plate and stained for caspase 3/7 activity (CellEvent™ Caspase 3/7 detection reagent; Invitrogen), subsequently the entire wells were imaged at an excitation maximum of 488 nm at 10× magnification in a Cell Discoverer 7 (Carl Zeiss). Images were processed using the Fiji software (ImageJ) and cells were quantified using the Particle Analyser tool.

### Western blot analysis

Differentiated IECs were colonized with *L. rhamnosus* or *L. brevis* and infected with *C. albicans* for 6, 12 and 24 h (see Table S1). Following incubation, supernatants were removed and cells were scraped off in PBS followed by centrifugation for 1 min at 500 ***g***. The pellet was resuspended in 100 μl modified RIPA buffer [50 mM Tris-HCl (pH 7.4), 150 mM NaCl, 1 mM EDTA, 1% Triton X-100, 1% sodium deoxycholate, 0.1% SDS] containing protease inhibitors (cOmplete Protease Inhibitor Cocktail, Roche) and phosSTOP phosphatase inhibitor cocktail tablets (Roche). Lysates were cleared by centrifugation for 5 min at 20,000 ***g*** at 4°C and protein concentration was quantified using a BCA assay kit (Thermo Fisher Scientific) according to the manufacturer's instructions. Diluted protein extracts were denatured in 1× Laemmli Sample Buffer [125 mM Tris-HCl (pH 6.8), 50% glycerol, 4% SDS, 0.02% Bromphenol blue, 0.1% 14 M 2-mercaptoethanol] for 5 min at 95°C and centrifuged for 5 s at 5000 ***g***. For SDS-PAGE, 30 µg of sample was separated on 10% SDS PAGE gels using a Mini-PROTEAN^®^ Tetra Cell system (Bio-Rad) and separated proteins were transferred to a nitrocellulose membrane (Amersham^TM^ Proton^TM^ 0.45 μM NC, GE Healthcare). The membranes were blocked with 5% I-Block protein-based blocking reagent (Life Technologies), solved in TBS-T [50 mM Tris-HCl (pH 7.6), 0.15 M NaCl, 0.05% Tween-20] and then incubated with primary antibodies E-cadherin (1:500, goat anti-human, AF648, R&D Systems) and GAPDH (1:500, rabbit anti-human, NB300-327, Novus) o/n at 4°C. After washing three times with TBS-T, membranes were incubated with a horseradish peroxidase-conjugated secondary antibody (E-Cadherin: anti-goat, 1:2000, SC-2020, Santa Cruz Biotechnology; GAPDH: anti-rabbit, 1:500, P0217, Dako) followed by three washing steps. Immunoreactivity was detected using enhanced chemiluminescence (ECL Plus Western Blotting Substrate, Thermo Fisher Scientific). Coomassie staining of membrane or gel was used to ensure equal loading of supernatant samples to the gel (not shown).

### Statistical analyses

Experiments were performed in biological triplicates (*n*≥3) unless otherwise stated. Data are mean of biological replicates±s.e.m. Data were analyzed using GraphPad Prism 7. For significance testing, lognormal ratio values were log transformed and tested against deviation from zero using a two-tailed *t*-test or by means of a one-way analysis of variance (ANOVA) test with a follow-up test for multiple comparisons (Tukey's correction). Other values were tested using two-tailed *t*-tests against the reference condition. Normality of individual distributions were ascertained by Shapiro-Wilk normality tests (threshold *P*>0.05) before claiming significance by *t*-tests, one-way ANOVA or two-way ANOVA. Statistical signiﬁcance is indicated in the graphs: *P≤0.05, **P≤0.01 or ***P≤0.001.

## Supplementary Material

Supplementary information
