# Supplementary material for: Keeping Candida commensal: how lactobacilli antagonize pathogenicity of Candida albicans in an in vitro gut model
Source: Dis Model Mech. 2019 Sep 12;12(9):dmm039719. doi: 10.1242/dmm.039719 (PMC6765188; doi:10.1242/dmm.039719)
Supplement: Supplementary information [file dmm-12-039719-s1.pdf]

Supplement

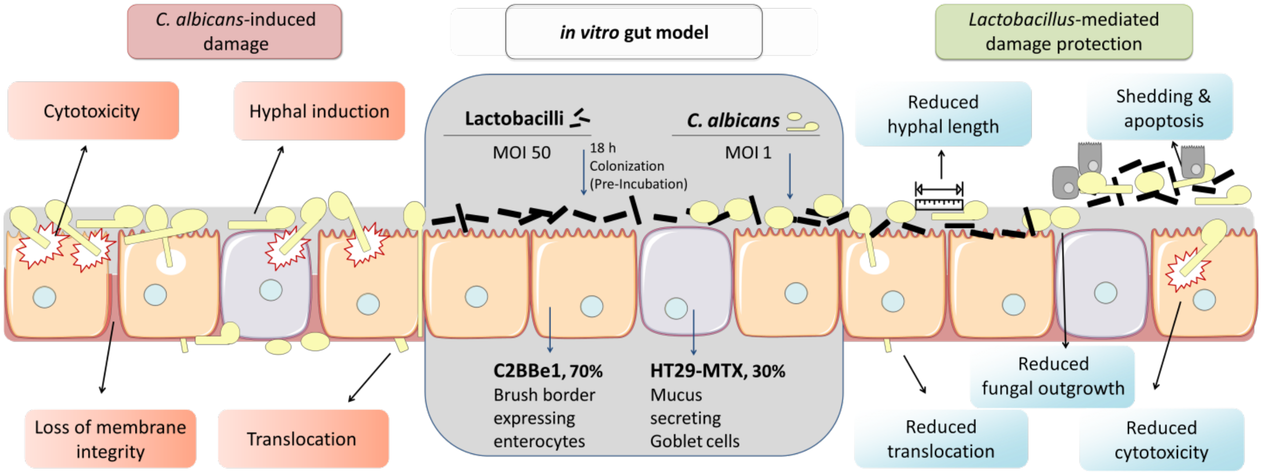

Figure S1: graphical abstract to visualize the written abstract.

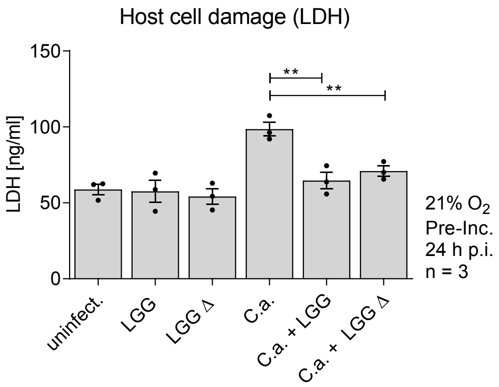

Figure S2: The protective effect of *L. rhamnosus* is independent of exopolysaccharides. LDH release at 24 h post-infection of IECs left untreated, colonized either with the *L. rhamnosus* GG mutant ((LGGΔ) lacking exopolysaccharides), or the corresponding WT strain (LGG) and infected with *C. albicans* (MOI 1) or not. The results were normalized to *C. albicans* single infection. Results shown are the mean values ± SEM, **p* < 0.05, ****p* < 0.005.

Disease Models & Mechanisms • Supplementary information

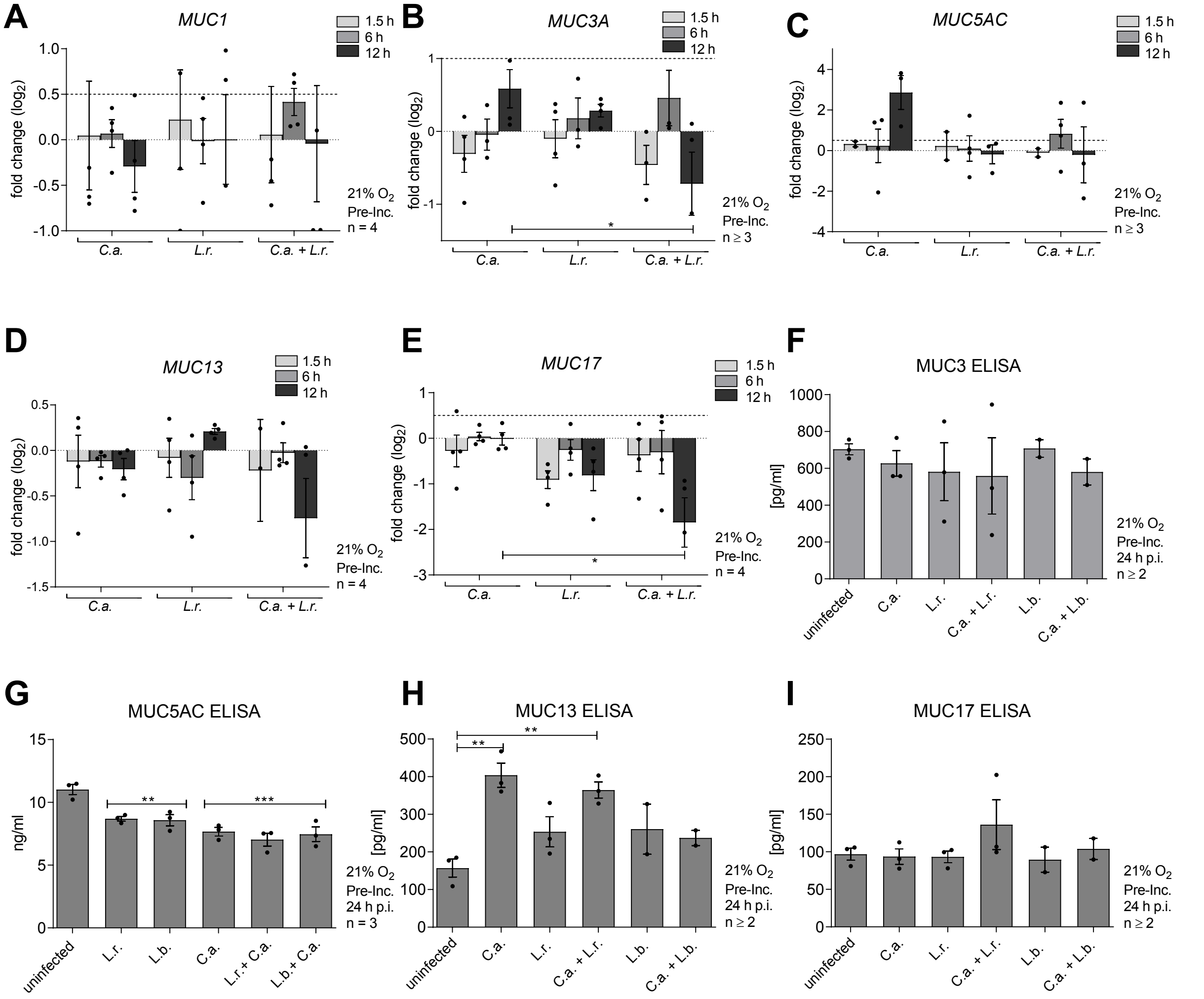

Figure S3: The protective effect of lactobacilli does not correlate with altered mucus secretion. The relative mRNA expression of (A) *MUC1*, (B) *MUC3A*, (C) *MUC5AC*, (D) *MUC13*, and (E) *MUC17* in IECs either left untreated or colonized with *L. rhamnosus* (MOI 50) and infected or not with *C. albicans* (MOI 1) for 1.5, 6, or 12 h. Expression levels were normalized to the reference genes *ACT1* and *GAPDH*. Results shown are the mean values ± SEM. **p* < 0.05, ***p* < 0.01, ****p* < 0.005. (F-I) Protein concentrations of (F) MUC3, (G) MUC5AC, (H) MUC13, and (I) MUC17 quantified in whole cell lysate of IECs left untreated or colonized with lactobacilli (*L. rhamnosus* or *L. brevis*; MOI 50) and infected or not with *C. albicans* for 24 h. Results shown are the mean values ± SEM. **p* < 0.05, ***p* < 0.01, ****p* < 0.005 compared to uninfected host cells.

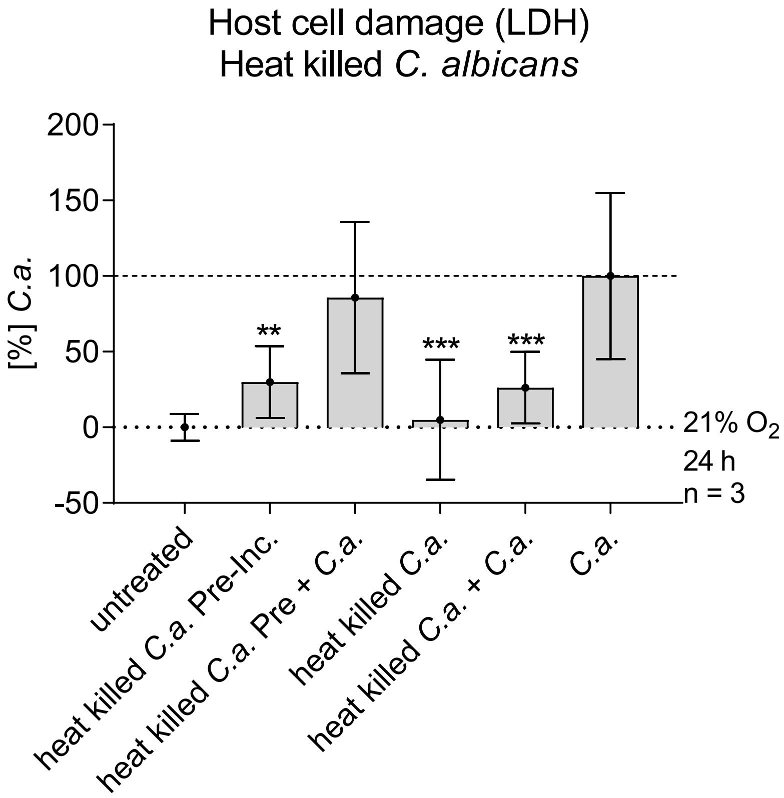

Figure S4: Protective effect through high biomass of heat-killed *C. albicans*. LDH release at 24 h post-infection of IECs colonized or not with heat-killed *C. albicans* (MOI 100) and infected with *C. albicans* (MOI 1) or not. The results were normalized to *C. albicans* single infection.

Experiment	*Format*	*initial No of IECs/well (volume)*	*CFU C.a./well (volume)*	*CFU lactobacilli/well [excess compared to C.a.] (volume)*	*total medium*	*incubation period*
Cytotoxicity (LDH assay)	96 well plate	2×10^4 (50 µl)	2×10^4 (50 µl)	1×10^5 [5x] (50 µl)<br>1×10^6 [50x] (50 µl)<br>5×10^6 [250x] (50 µl)	150 µl	24 or 48 h
Adhesion of *C. albicans*	24 well plate coverslips	1×10^5 (125 µl)	5×10^4 (125 µl)	2.5×10^6 [50x] (125 µl)	375 µl	1 h
Hyphal length	24 well plate coverslips	1×10^5 (125 µl)	5×10^4 (125 µl)	2.5×10^6 [50x] (125 µl))	375 µl	4 h
Translocation, TEER	transwell inserts	2×10^4 (50 µl)	2×10^4 (50 µl)	1×10^6 [50x] (150 µl)	250 µl	24 h
cell-free supernatants	96 well plate	2×10^4 (0 µl)	2×10^4 (10 µl)	-<br>undiluted SN 140 µl	150 µl	24 h
killed lactobacilli (pre-incubation)	96 well plate	2×10^4 (50 µl)	2×10^4 (50 µl)	1×10^6 [50x] (50 µl)	150 µl	24 h
killed lactobacilli (simult. infection), displacement	96 well plate	2×10^4 (50 µl)	2×10^4 (50 µl)	1×10^6 [50x] (50 µl)<br>5×10^6 [250x] (50 µl)<br>1×10^7 [500x] (50 µl)	150 µl	24 h
Growth of lactobacilli in IECs	24 well plate	1×10^5 (125 µl)	5×10^4 (0 µl)	$2.0\text{x}10^3$ [-10x] (1000 µl]	1000 µl	24 h
Western Blot	6 well plate	4×10^5 (1300 µl)	4×10^5 (1300 µl)	2.0×10^7 [50x] (1300 µl)	3900 µl	6 h, 12 h, 24 h
Glucose- and Lactate measurement	6 well plate	4×10^5 (1300 µl)	4×10^5 (1300 µl)	2.0×10^7 [50x] (1300 µl)	3900 µl	0 h, 6 h, 12 h, 24 h
C.a. shedding	6 well plate	4×10^5 (1300 µl)	4×10^5 (1300 µl)	2.0×10^7 [50x] (1300 µl)	3900 µl	1 h, 3 h, 6 h, 24 h
SEM	24 well plate coverslips	1×10^5 (125 µl)	5×10^4 (125 µl)	2.5×10^6 [50x] (125 µl)	375 µl	6 h
ELISA	24 well plate	1×10^5 (125 µl)	5×10^4 (125 µl)	2.5×10^6 [50x] (125 µl)	375 µl	24 h
RNA isolation	6 well plate	4×10^5 (1300 µl)	4×10^5 (1300 µl)	2.0×10^7 [50x] (1300 µl)	3900 µl	1.5 h, 6 h, 12 h
Apoptosis	96 well plate	2×10^4 (50 µl)	2×10^4 (50 µl)	1×10^6 [50x] (50 µl)	150 µl	0 h-24 h

Table S1: Overview of the exact number of host cells, *Lactobacillus* species, *C. albicans*, and incubation periods used in the respective experiments.

**target gene**	**sequence**	**target gene**	**sequence**
β-ACTIN	F: AAATGCTTCTAGGCGGACTAT<br>R: AAGGGACTTCCTGTAACAACG	*MUC13*	F: GAGCTTGCAACCTAGCCTCA<br>R: AGAGTGCACCCCATAGTGGA
GAPDH	F: TTGCCCTCAACGACCACTTT<br>R: GGTGGTCCAGGGGTCTTACT	*MUC17*	F: TTGTCAGCACCACACTTCCA<br>R: GGCAGTGTCAGTAGAAGGGG
MUC1	F: CAGTGCCGCCGAAAGAACTA<br>R: TAGGGGCTACGATCGGTACT	*cFOS*	F: GGAGGAGGGAGCTGACTGAT<br>R: GCTGCCAGGATGAACTCTAGT
MUC2	F: CACTGCCCTCGACAGCTTTA<br>R: TGGAAAGCAAGGACTGAACAA	*DUSP1*	F: CTGCAGTTTGAGTCCCAGGT<br>R: AGACGGGGAAGTTGAACACG
MUC3A	F: TCACATCCTGGTCCCTAGCA<br>R: TGAATGGACGGGCTCAGAAG	*DUSP5*	F: TGGAAGACAGCCACACGG<br>R: GAGATCCCAGCCTCACAGTG
MUC4	F: CAGCTCCTGGGCTGAACATT<br>R: CTCGTGTGAAGTCCGATGCT	*NFκB-IA*	F: ACCTCACCTTTGTGGGGTTT<br>R: ACAGGATACCACTGGGGTCA
MUC5AC	F: GTACTCGCTCGAGGGCAACA<br>R: TTCCACCTCGGTGTAGCTGA		

Table S2: Primers used for gene expression analyses

Disease Models & Mechanisms • Supplementary information
